# Locus‐Specific Convergent Evolution and Interchromosomal Rearrangements Contribute to the Diversification of Amniote Type I Interferons

**DOI:** 10.1111/eva.70258

**Published:** 2026-05-14

**Authors:** Le Zhang, Fubo Ma, Jinpeng Liu, Yangchao Yu, Junxiao Ma, Lei Zhang, Bing Li, Chaofan Li, Kang Li, Peng Liu, Liguo Zhang

**Affiliations:** ^1^ College of Computer Science Sichuan University Chengdu China; ^2^ West China Biomedical Big Data Center West China Hospital, Sichuan University Chengdu China; ^3^ Beijing Zoo Beijing China; ^4^ University of Chinese Academy of Sciences Beijing China; ^5^ Hebei Key Laboratory of Analysis and Control of Zoonotic Pathogenic Microorganism, Hebei Wild Animal Health Center Hebei Agricultural University Baoding China; ^6^ Key Laboratory of Biomacromolecules (CAS), National Laboratory of Biomacromolecules, CAS Center for Excellence in Biomacromolecules, Institute of Biophysics Chinese Academy of Sciences Beijing China

**Keywords:** common ancestor, evolution, gene diversification, type I interferons

## Abstract

Type I interferons (IFNs) play essential roles in antiviral immune responses. The extensive diversification of type I IFNs into various subtypes and duplicated gene copies has posed significant challenges for evolutionary reconstruction. To address this, we developed the type I IFN sequence composition and structure (IFN‐SCOPE) model and gene‐network graph degree centrality (GENE‐GRADE) algorithm, which transform the discovery of type I IFN evolutionary trajectories into computing the node centrality in its gene networks. Through synteny‐guided analysis, we verified that three previously reported evolutionarily conserved type I IFN loci (HACD4, MOB3B, and UBAP2) have maintained chromosomal colocalization across all major amniote lineages. While the MOB3B locus maintained a single IFN‐κ ortholog, the HACD4 (IFN‐HA) and UBAP2 (IFN‐UB) loci showed lineage‐specific expansion patterns: IFN‐HA proliferated in mammals/reptiles but remained single‐copy in birds, whereas IFN‐UB expanded in birds but not in other lineages. A phylogenetic analysis revealed that these independently evolved multicopy genes nevertheless clustered into two conserved subgroups (IFN‐HA2/HA1 and IFN‐UB2/UB1), suggesting convergent functional specialization. Within the IFN‐HA and IFN‐UB clusters, the single‐copy IFN‐HA2 and IFN‐UB2 genes, positioned at the ancestral ends of their respective genomic arrays, likely represent the progenitor sequences of each locus, where the poorly characterized IFN‐ν (rather than IFN‐β) is the ancestral form of mammalian IFN‐HA subtypes. Furthermore, wet lab evidence revealed type I IFN genes at noncanonical loci resulting from interchromosomal duplication events in tortoises and diving ducks and provided clear evidence that interchromosomal duplications contributed to type I IFN gene diversity. These discoveries advance our understanding of the evolutionary mechanisms that shape type I IFN genes in amniotes and are potentially beneficial for the development of novel type I IFN‐based antiviral treatments through comparative immunological approaches.

## 1 Introduction

Type I interferons (IFNs) are a family of cytokines that can be induced in almost all lineages of cells upon pathogen infection (Borden 2019; Ivashkiv and Donlin 2014). Owing to their antiviral properties, type I IFNs have been developed into therapeutic agents for treating viral infections or cancer (Wittling et al. 2020; Mueller and Hartmann 2021; Fox et al. 2020). On the other hand, prolonged type I IFN signaling can be pathogenic and drive autoimmune diseases, including systemic lupus erythematosus (SLE) (Crow and Stetson 2021; Crow 2022). Consequently, the evolution of type I IFNs requires a delicate balance to maintain effective protection without causing excessive detrimental effects.

Type I IFN genes originated in jawed vertebrates and have diversified extensively across different types of vertebrate lineages (Krause and Pestka 2015; Secombes and Zou 2017). While type I IFN genes of both cartilaginous fish and bony fish contain introns, amphibians possess both intron‐containing and intronless variants (Gan et al. 2017, 2018; Adeyemi et al. 2023). In contrast, amniote type I IFNs arose from a single retroposition event during the adaptation of tetrapods to terrestrial life (Secombes and Zou 2017; Gan et al. 2018; Qi et al. 2010; Chen et al. 2021). Thus, the term “intronless” type I interferons (intronless with single quotation marks) has been proposed to describe type I IFNs in all amniotes, which focuses on their evolutionary origins, although some amniote type I IFNs have since reintroduced introns (Chen et al. 2021, 2022). In humans, the type I IFN family comprises IFN‐α (with 13 subtypes), IFN‐β, IFN‐ω, IFN‐ε, and IFN‐κ, each of which exhibits functional diversity because of their differences in receptor affinity, induction pathways, or tissue specificity (Wittling et al. 2020; Klein et al. 2020; Bourke et al. 2022). While these common subtypes are conserved across many mammals, lineage‐specific subtypes such as IFN‐αω, IFN‐δ, IFN‐τ, IFN‐ζ, and IFN‐ν have also independently evolved (Krause and Pestka 2015, 2005), likely driven by selective pressures from diverse viral infections in different animals (Schreiber and Piehler 2015).

The genes encoding type I IFNs in the analyzed mammals were shown to primarily form a single cluster (locus a) between HACD4 and MTAP, with the exception of IFN‐κ, which was found at a distinct locus (locus b) between MOB3B and C9ORF72 (Krause and Pestka 2015). A homologous gene at locus b has been found in sauropsids and is considered a true ortholog of mammalian IFN‐κ, although it is absent in some species, such as chicken ( *Gallus gallus* ) and the Chinese soft‐shelled turtle ( *Pelodiscus sinensis* ) (Krause and Pestka 2015; Chen et al. 2022; An Ning Pang et al. 2025). Beyond these two loci, sauropsids possess an additional type I IFN locus (locus c) within an intron of the UBAP2 gene. At locus a, reptilian IFNs are divided into two phylogenetic subgroups: a single‐copy IFN‐III (IFN‐C) and a multicopy IFN‐IV (IFN‐D) (Krause and Pestka 2015; Chen et al. 2022). In contrast, birds possess only a single‐copy gene (IFN‐III) at locus a. While initially classified as an IFN‐κ homolog on the basis of phylogeny (Santhakumar, Iqbal, et al. 2017; Gao et al. 2018), subsequent syntenic and functional analyses have proposed designating it as IFN‐κ‐like (IFNKL) (Santhakumar, Rubbenstroth, et al. 2017). The genes at locus c have been functionally classified in birds as “IFN‐α” and “IFN‐β” because their characteristics resemble those of their mammalian counterparts: avian IFN‐β is a single‐copy gene and contains an NF‐κB consensus motif in its promoter, whereas avian IFN‐α is a multicopy gene and responds to resiquimod stimulation (Sick et al. 1998; Lowenthal et al. 2001). Although these are not bona fide orthologs of mammalian IFNs on the basis of synteny, the nomenclature remains in use owing to these functional parallels. In contrast to birds, reptiles possess only a single‐copy IFN gene at locus c (Krause and Pestka 2015; Chen et al. 2022).

Previous functional studies in geese, chickens, and Chinese soft‐shelled turtles have confirmed that sauropsid type I IFNs possess potent antiviral activity, underscoring their fundamental functional similarity to their mammalian counterparts (Chen et al. 2022; Santhakumar, Iqbal, et al. 2017; Santhakumar, Rubbenstroth, et al. 2017; Jiang et al. 2011). Currently, our understanding of functional differences between type I IFN subtypes is largely derived from studies in humans and mice, with limited data from pet and farm animals and a near‐total absence from other species. Therefore, the comparative immunology of type I IFNs across diverse taxa is crucial to elucidate their evolutionary trajectory and functional diversification. However, the phylogenetic ambiguity and rapid sequence divergence of type I IFNs among amniotes complicate the identification of true orthologs across these diverse species (Krause and Pestka 2015, 2005; Secombes and Zou 2017).

In this study, we present an integrated bioinformatics pipeline combining the type I IFN sequence composition and structure (IFN‐SCOPE) model and a gene‐network graph degree centrality (GENE‐GRADE) algorithm to comprehensively identify type I IFN coding sequences and analyze their genomic context. Using a synteny‐guided phylogenetic approach, we systematically investigated the evolution of type I IFNs in amniotes.

## 2 Results

### 2.1 Identification of 115 Unannotated Type I IFNs by the IFN‐SCOPE Model

Frequent duplication and rapid diversification lead to low sequence homology of type I IFN genes between evolutionarily distant animals or even between different type I IFN subtypes in the same animal species. The low sequence homology of type I IFNs hinders the complete and accurate annotation of type I IFN genes. Therefore, we developed a computational method based on BLAST (Altschul et al. 1990) and a custom IFN‐SCOPE model to systematically identify unannotated type I IFN genes in candidate species.

First, we extracted 793 annotated type I IFN genes from 87 amniotes (Figure S1). These type I IFNs included 56 IFN‐α and 15 IFN‐β (in mammals), 24 IFN‐κ (in mammals and birds), 12 IFN‐ε (in mammals), 11 IFN‐δ (in sheep), 10 IFN‐ω (in humans, artiodactyls and chickens), 7 IFN‐τ (in ruminants), 1 IFN‐ζ (in mice), 14 type I IFNs designated with “Gm” (such as Gm13282, in mice), 5 type I IFNs designated with numbers (such as IFN1 in platypus and sheep), and 638 unclassified type I IFN genes identified with “LOC” numbers. All these type I IFN genes had amino acid sequences provided by public databases (Krause and Pestka 2015).

Next, we used the amino acid sequences of annotated type I IFNs as query sequences and obtained potential protein‐coding regions of type I IFN on the genomes of candidate species by BLAST. Afterward, we entered the newly identified sequences into the IFN‐SCOPE model to obtain predictive results for unannotated type I IFN. A total of 793 positive samples (i.e., amino acid sequences of IFN) and 2041 type I IFN‐adjacent genes were extracted from the training dataset after data preprocessing.

Afterward, we carried out feature selection on the basis of the amino acid composition and the hydrophobicity and the hydrophilicity correlations between residues of sample sequences. We subsequently conducted feature dimensional reduction to decrease the overfitting risk and improve the model performance. As presented, the feature space was reduced from 80 dimensions to 36 dimensions after dimensional reduction (Table S1). Matrices and corresponding sample labels were subsequently entered into a logistic regression classifier for model training, and we evaluated the performance of the model before and after feature dimensional reduction by various metrics of 5‐fold cross‐validation (Table S1). All of the model metrics improved after feature dimensional reduction, which demonstrated the effectiveness of the procedure.

Finally, we entered the test set constructed by BLAST to our well‐trained logistic regression model to obtain the prediction results for unannotated type I IFNs. A total of 115 unannotated type I IFNs were discovered by the IFN‐SCOPE model (Table S2).

### 2.2 Three Conserved Type I IFN Loci Determined by GENE‐GRADE Are Located on the Same Chromosome

**FIGURE 1:**
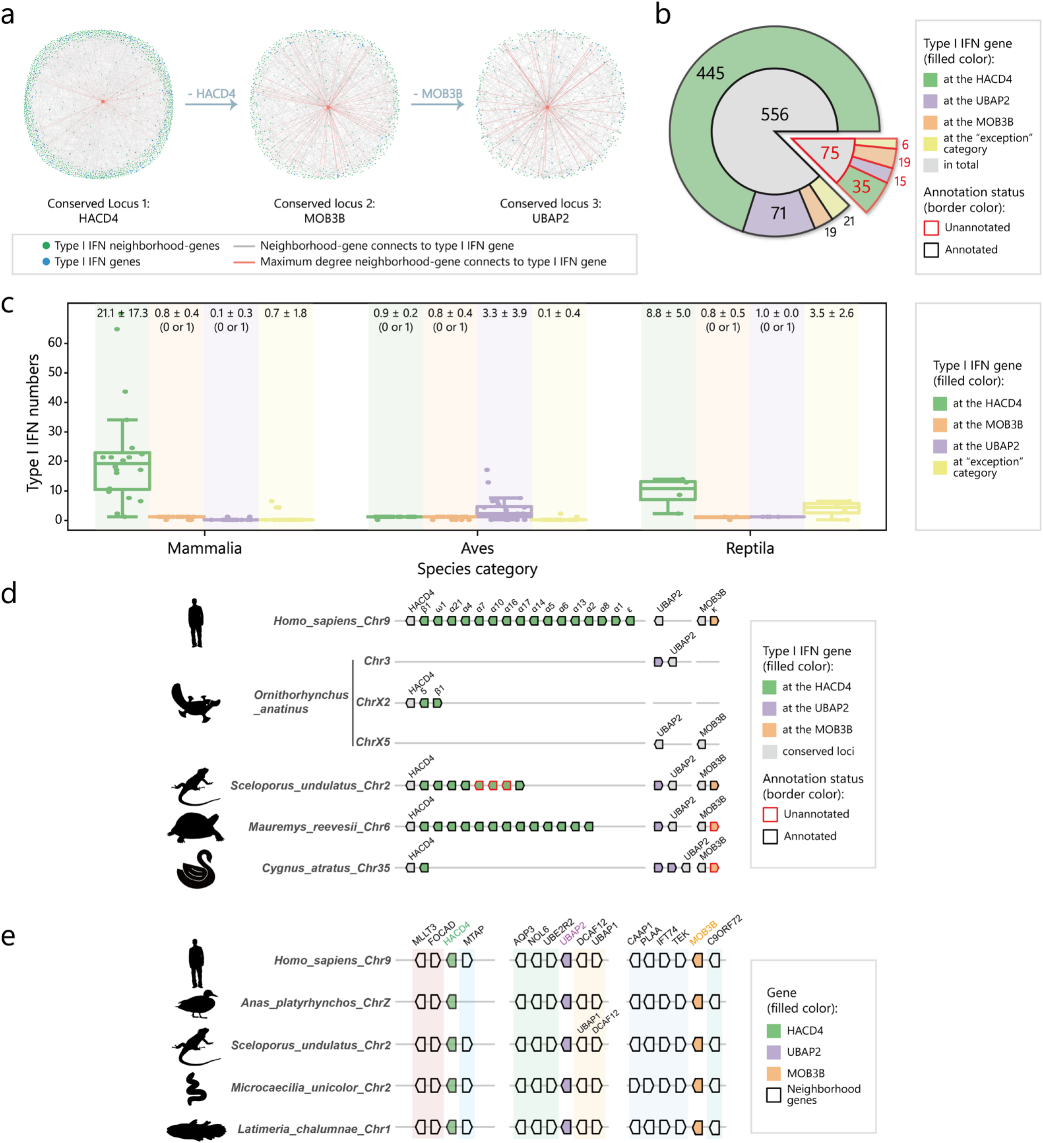
Conserved loci determined by the GENE‐GRADE algorithm. (a) The results of the GENE‐GRADE algorithm. Each subplot represents the knowledge graph before output of the conserved loci HACD4, MOB3B, and UBAP2. The gray lines represent the connections between nodes in the knowledge graph, whereas the orange lines represent the connections between the maximum degree node (the node of the conserved locus) and other nodes. (b) The numbers of placed type I IFN genes in the conserved loci or “exception” category. Type I IFN genes were assigned as annotated or unannotated in the pie chart, and their corresponding numbers are shown in the inner circle of the pie chart, while the outer circle illustrates the numbers of type I IFN genes at the HACD4 locus, UBAP2 locus, MOB3B locus, and “exception” category. (c) Box plots of the numbers of type I IFN genes at conserved loci or “exception” categories for species in mammalia, aves, and reptilia. The label on the top of the diagrams represents the mean and standard deviation of the corresponding boxes, and “(0 or 1)” was added after the statistical value if there was only a single‐copy type I IFN gene or no type I IFN gene at the corresponding locus. (d) The distribution of all type I IFN genes at conserved loci or “exception” categories in representative species. Type I IFN genes on the chromosomes of humans ( *Homo sapiens* ), platypus ( *Ornithorhynchus anatinus* ), fence lizards ( *Sceloporus undulatus* ), turtles ( *Mauremys reevesii* ), and black swans ( *Cygnus atratus* ) are displayed according to their corresponding conserved loci. (e) The distribution of three conserved loci and their neighborhood genes in four species. Conserved neighborhood genes are marked by different color shadings. Type I IFN genes are not shown, and the gene orientations are adjusted to align the gene orientations in humans in this figure.

To overcome the challenges posed by the high sequence divergence of type I IFN genes, we developed the GENE‐GRADE algorithm. Its objective was to quantify and evaluate the conservation of genes neighboring each type I IFN gene by applying a degree centrality algorithm (Ghasemi et al. 2014), thereby enabling the identification of conserved type I IFN genes across species. In this study, we included 556 annotated and 75 unannotated type I IFN genes, which are genes that were placed onto specific chromosomes (unplaced genome scaffolds were excluded) of the candidate species. Three conserved loci, HACD4, MOB3B, and UBAP2, were output by the GENE‐GRADE algorithm (Figure 1a). Although these three loci have been previously reported (Krause and Pestka 2015; Chen et al. 2022), we have confirmed these findings through an objective and data‐rich computational perspective.

The type I IFN genes located at these three conserved loci constituted 95.7% (604 out of 631) of all type I IFN genes in our study (Figure 1b), and the majority of them localized to the HACD4 locus (445 annotated and 35 unannotated), while fewer resided at UBAP2 (71 annotated and 15 unannotated) and MOB3B (19 annotated and 19 unannotated). The remaining 27 genes (21 annotated and 6 unannotated) were assigned to uncharacterized loci (“exception” category) or required further validation.

Interestingly, the numbers of type I IFN genes at the 3 conserved loci varied among the different animal groups (Figure 1c, Figures S2, S3 and Table 1). In all the animals analyzed, type I IFN genes were monogenic or absent at the MOB3B locus (Figure 1c,d, Table 1). While the type I IFN genes at the HACD4 locus were monogenic in birds, they were polygenic in mammals (median: 21) and reptiles (median: 9), including turtles, crocodilians, and squamates (Figure 1c,d). Conversely, type I IFN genes at the UBAP2 locus were monogenic in mammals and reptiles but polygenic in birds (median: 3) (Figure 1c,d), but in mammals, only monotremes (platypus and echidna) had a single‐copy type I IFN gene at the UBAP2 locus (Figure 1d, Figure S2).

**TABLE 1:** The numbers of type I IFN genes in the conserved loci.

Conserved loci	Mammalia	Aves	Reptila
HACD4	MC	SC	MC
UBAP2	SC (monotremes) NF (most mammals)	MC	SC
MOB3B	SC	SC	SC

Abbreviations: MC, multiple‐copy; NF, not found; SC, single‐copy.

The DNA fragments containing these 3 conserved type I IFN loci (HACD4, MOB3B and UBAP2) were located on the same chromosome in most of the animals we analyzed, with few exceptions, including pig ( *Sus scrofa* ), common brushtail possum ( *Trichosurus vulpecula* ), platypus ( *Ornithorhynchus anatinus* ) and echidna ( *Tachyglossus aculeatus* ) (Figure 1d, Figures S2 and S3). Importantly, the conserved colocalization of HACD4, MOB3B, and UBAP2 on a single chromosome predated the integration of “intronless” type I IFN genes, as evidenced in unstriped caecilian ( *Microcaecilia unicolor* ) and coelacanth ( *Latimeria chalumnae* ) (Figure 1e). Collectively, these observations support the hypothesis that “intronless” type I IFN genes were already integrated at the HACD4, MOB3B, and UBAP2 loci in the most recent common ancestor (MRCA) of modern amniotes.

### 2.3 Both Evolutionary Trajectories and Locus Specificity Contribute to the Complexity of Type I IFN Phylogeny

**FIGURE 2:**
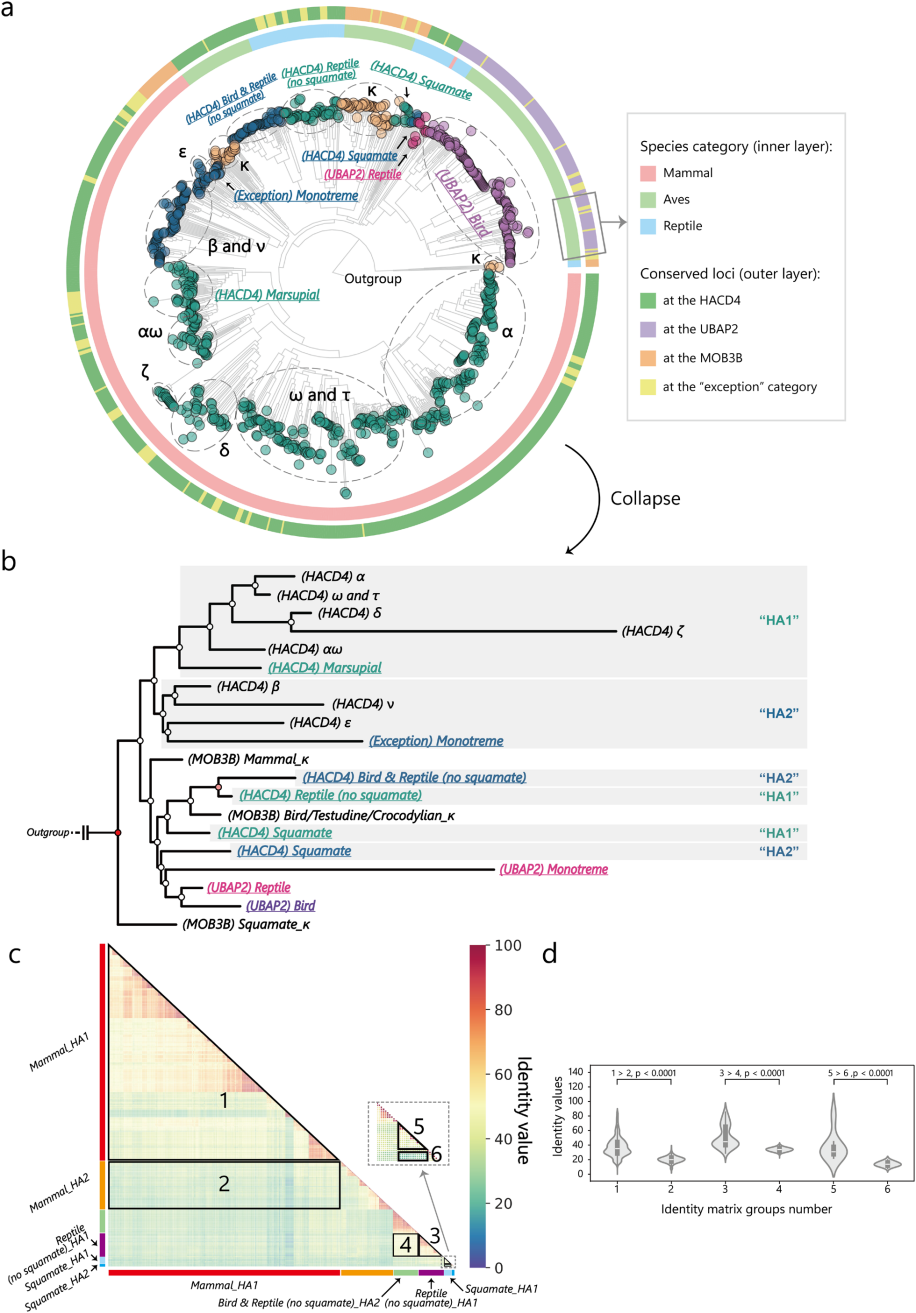
The phylogenetic relationships of amniote type I IFN genes in this study (a). Amniotes type I IFN genes were assigned to the following clades: HA1 (dark green), HA2 (dark blue), κ (brown), UB1 (dark purple), and UB2 (light purple). The layers surrounding the tree represent the corresponding species category and the conserved loci, respectively. First‐reported type I IFN clades are highlighted by underlining and named by conserved loci and species category in the figure. (b) The phylogenetic tree that collapsed according to the type I IFN clades shown in (a). The order of the taxa in the phylogenetic tree, from top to bottom, corresponds to the clockwise order of the tree taxa in (a). (c) The sequence identity matrix of IFN‐HAs in candidate species. The grouping of taxa is indicated by color bars. (d) Violin plots of the identity values in the six regions in (c) and the results of the significance test.

We performed a phylogenetic analysis of the collected amniote type I IFN genes and plotted the data with both species and locus information. Surprisingly, we found that the phylogenetic tree displayed a mosaic pattern, reflecting a mixture of evolutionary history and locus specificity (Figure 2a,b).

Specifically, almost all type I IFN genes at the UBAP2 locus (IFN‐UB) were clustered together (Figure 2a,b). The type I IFN genes at the MOB3B locus were phylogenetically classified into three distinct lineages: mammals, squamates, and Archelosauria (including turtles, crocodilians, and birds) (Figure 2b). A parallel evolutionary pattern is observed at the HACD4 locus, where these type I IFN genes (IFN‐HA) similarly diverged into these three lineages.

Notably, each lineage‐specific IFN‐HA group further diversified into two subclusters: the first cluster of mammalian IFN‐HAs (mammalian IFN‐HA1; Figure 2b) comprised almost all multicopy subtypes (IFN‐α, IFN‐ω, IFN‐τ, IFN‐αω, IFN‐δ, and IFN‐ζ) of placental mammals (Wittling et al. 2020; Krause and Pestka 2015; Secombes and Zou 2017; Xu et al. 2013), as well as multiple‐copy type I IFN genes of marsupials. The second cluster of mammalian IFN‐HAs (mammalian IFN‐HA2, Figure 2b) included IFN‐β, IFN‐ν, and IFN‐ε of therian mammals, along with all type I IFN genes of monotremes (Krause and Pestka 2015). Correspondingly, the Archelosaurian IFN‐HA1 and IFN‐HA2 clusters were located adjacent to each other in the phylogenetic tree. The division of the IFN‐HA1 and IFN‐HA2 groups was further supported by the sequence identity matrix analysis. The identity values within IFN‐HA1s were greater than those between IFN‐HA1s and IFN‐HA2s in mammals (1 vs. 2), Archelosaurians (3 vs. 4), and squamates (5 vs. 6) (Figure 2c,d).

Surprisingly, although the squamate IFN‐HA1 cluster grouped with other IFN‐HAs (Figure 2a,b), the squamate IFN‐HA2 genes phylogenetically clustered with type I IFN genes at the UBAP2 locus (although their branch length indicated distance from other IFN‐UBs). This surprising phylogenetic placement was quantitatively supported by our sequence identity matrix analysis, in which the identity values between IFN‐HA1 and IFN‐HA2 in squamates were significantly lower than those within the IFN‐HA1 subtype (Figure 2c, 5 vs. 6, Figure 2d, *p* < 0.0001).

### 2.4 Evolutionarily Conserved IFNK Orthologs Across Amniotes

**FIGURE 3:**
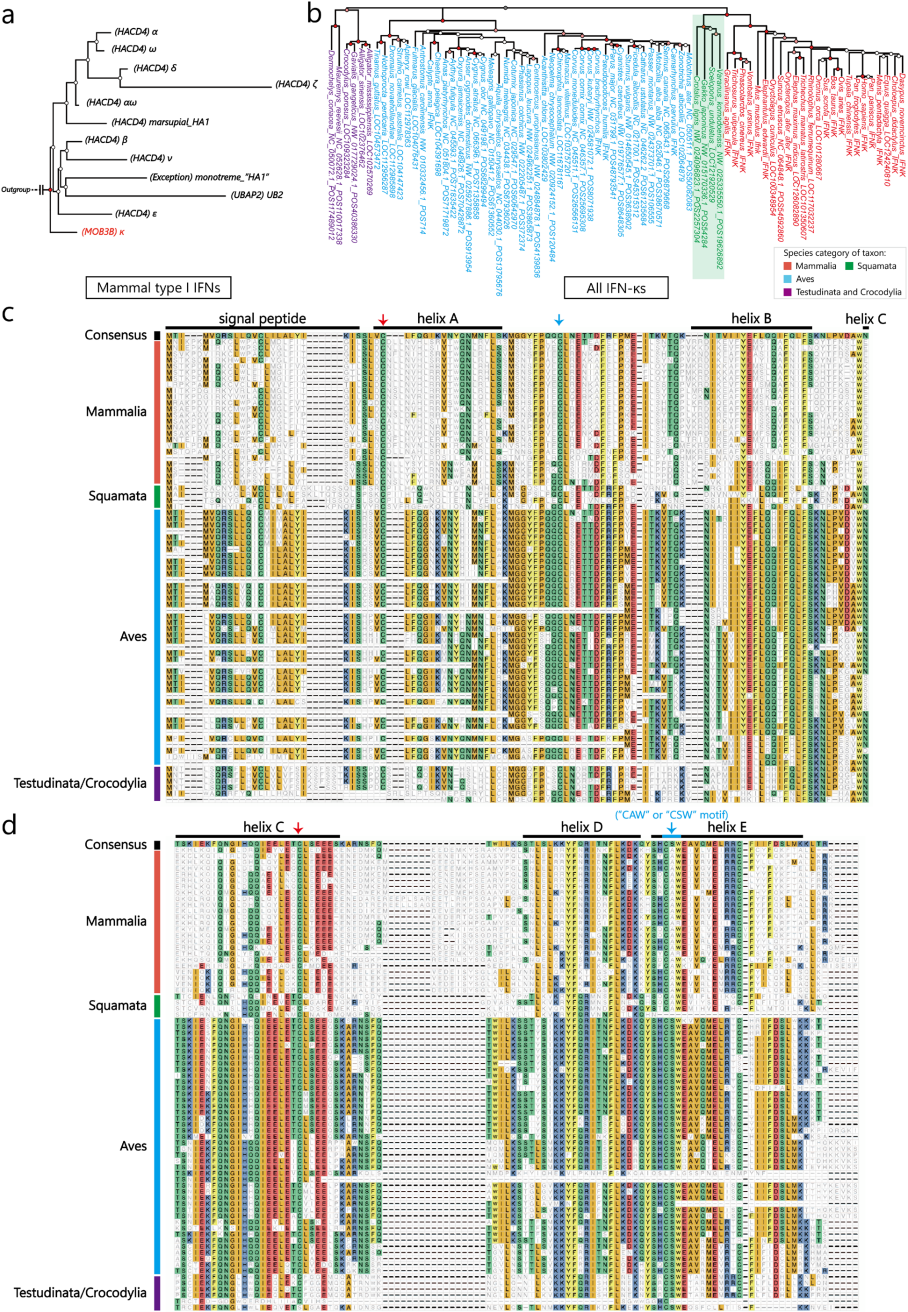
Evolution of IFN‐κ orthologs. (a) Phylogenetic relationships of all mammalian type I IFNs. The monophyletic group of IFN‐κs is colored in red. (b) The phylogenetic relationships of all IFN‐κs. Species are denoted with different colors: Mammalia (red), squamata (green), aves (blue), and testudinata & crocodylia (purple). (c) The first half of the MSA results of all IFN‐κs in our candidate species. (d) The second half of the MSA results of all IFN‐κs in our candidate species.

The MOB3B locus showed remarkable conservation, maintaining one type I IFN copy (or zero) throughout amniote evolution (Figure 1c, Figures S2, S3) and formed one single cluster among mammalian type I IFN genes (Figure 3a). In addition, neighborhood genes, such as C9ORF72, LINGO2, and TEK, were conserved in most amniotes (Figure S4a). Notably, all type I IFN genes at the MOB3B locus were co‐oriented and transcribed in the same direction as MOB3B (Figure S4a). On the basis of synteny analysis, the type I IFN genes at the MOB3B locus clearly represented orthologs of human IFNK (the gene encoding IFN‐κ) throughout amniotes (Figures S5, S6). Notably, the type I IFN gene at the HACD4 locus of chicken ( *Gallus gallus* ) and mallard ( *Anas platyrhynchos* ) has been classified as IFN‐κ through a conventional genome annotation pipeline in a public database (Santhakumar, Iqbal, et al. 2017; Gao et al. 2018; O'Leary et al. 2016). This method, which relies mainly on homology‐based evidence from available sequences, leads to mistakes stemming from the limitations in data updating and algorithm generalization performance (O'Leary et al. 2016). However, the three conserved loci, determined by the GENE‐GRADE algorithm, encouraged us to reconsider this nomenclature through a syntenic view. As a result, evidence from both of these animals revealed a truncated form of the IFNK sequence beside MOB3B, contradicting the nomenclature of type I IFN at the HACD4 locus of birds as orthologs of human IFNK (Figure S6).

Interestingly, our results revealed that the IFN‐κ of squamates (green in Figure 3b) formed a monophyletic group with mammalian IFN‐κ first and then grouped with the remaining avian and reptilian IFN‐κ. Moreover, the IFN‐κ of birds, testudines, and crocodylians differed significantly from that of mammals and squamates, as indicated by the filled color of the MSA results (Figure 3c). Additionally, around the fourth conserved cysteine (Secombes and Zou 2017) (blue arrow in Figure 3d), mammalian and squamate IFN‐κ shared a conserved “CAW” motif (cysteine‐alanine‐tryptophan), whereas avian, testudine, and crocodylian IFN‐κ exhibited a “CSW” motif (cysteine‐serine‐tryptophan). Thus, we propose that the major speciation event of IFN‐κ might have occurred during squamata–testudinata speciation rather than mammalia–sauropsida speciation, as indicated by taxonomy (Figure S1).

### 2.5 Two‐Subcluster Division of IFN‐HAs Is Conserved Between Mammals and Reptiles

**FIGURE 4:**
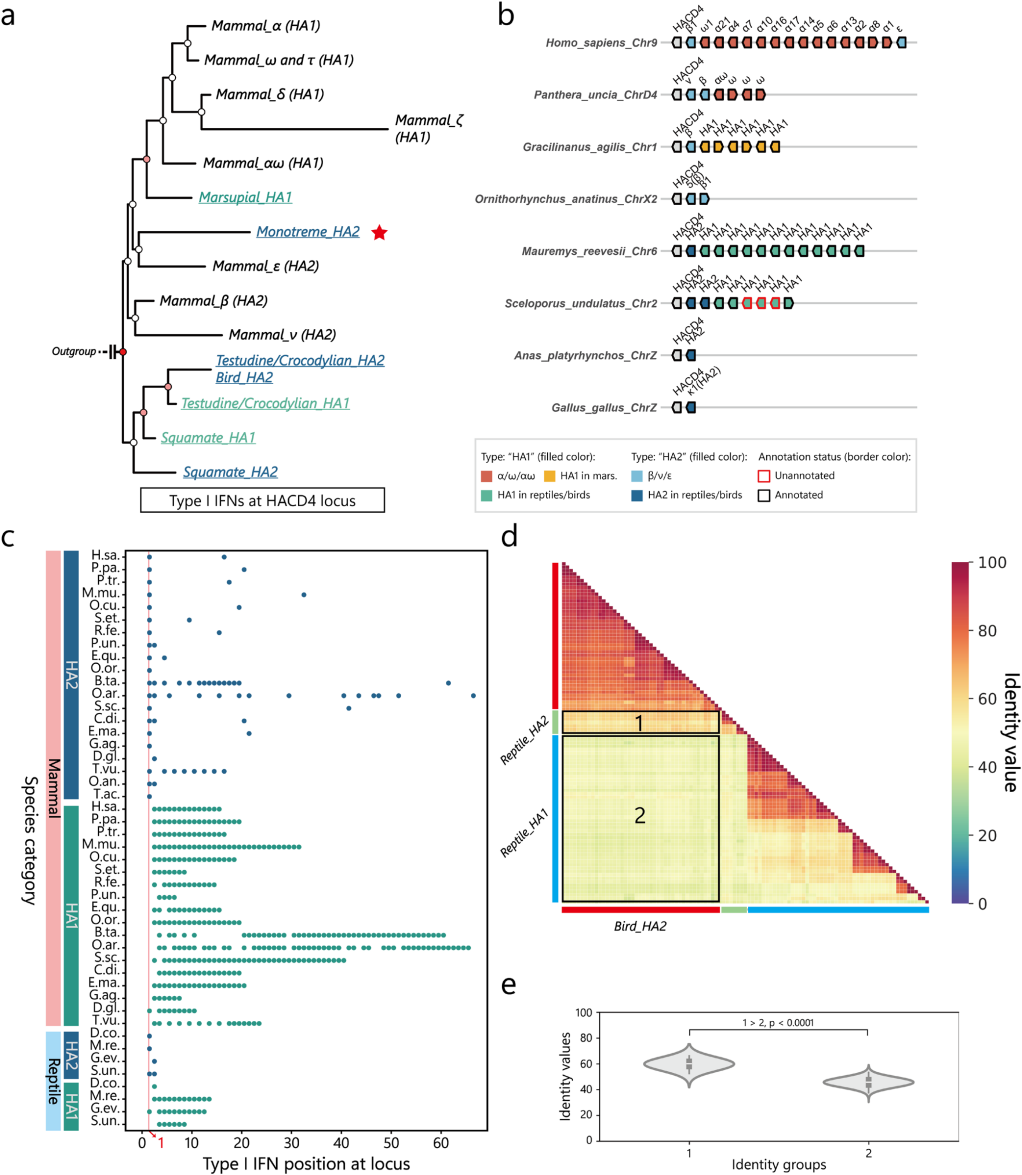
IFN‐HAs can be classified as HA1 and HA2. (a) Phylogenetic relationships of all IFN‐HAs. HA1 or HA2 are marked for every corresponding clade in the tree. First‐reported type I IFN clades are highlighted by color and underlining. A red star indicates the specific IFN‐HA2 clade in the monotremes. (b) The distribution of type I IFN genes at HACD4 on the chromosomes of representative species. Type I IFN subtypes can be classified into “HA1” and “HA2.” (c) The relative type I IFN position of IFN‐HAs. Each data point represents the position of a type I IFN gene at the HACD4 locus in the corresponding species. (d) The sequence identity matrix of the HA1 and HA2 genes in reptiles and birds. The grouping of taxa is indicated by color bars. (e) Violin plots of the identity values in the two regions in (d) and the results of the significance test.

Both mammals and reptiles possess multiple IFN‐HA genes, whereas birds contain a single IFN‐HA gene at the HACD4 locus (Figure 1, Table 1). Phylogenetic analysis of all IFN‐HA genes revealed that IFN‐HAs in sauropsids clustered together and formed a sister group to mammalian IFN‐HA2s (IFN‐β/ν/ε) and mammalian IFN‐HA1s (IFN‐α/ω/δ) (Figure 4a). Notably, the multiple‐copy monotreme IFN‐HA2 genes (Figure 4a, star‐labeled) were located on chromosome X5 (Figure S5), where they neighbor the TBC1D2 gene, which typically flanks MOB3B in other vertebrates (Figure S4a). However, unlike canonical IFNKs, they were not positioned between MOB3B and C9ORF72 (Figure S5). Phylogenetically, these genes clustered with IFN‐HAs rather than with IFNKs, suggesting their evolutionary origin as true IFN‐HA homologs. This unique genomic arrangement of monotreme IFN‐HA2s likely reflects the specialized ecological and microbial adaptations of these egg‐laying mammals (Zhou et al. 2021).

In mammals and reptiles, the IFN‐HA genes diverged into IFN‐HA1 and IFN‐HA2 subclusters (Figures 2, 4a). Notably, HACD4‐adjacent IFN‐HA genes were phylogenetically restricted to the IFN‐HA2 subcluster and in the same transcriptional orientation (Figure 4b,c). Further analyses revealed a consistent pattern across species: the HACD4‐adjacent IFN‐HA2 gene was typically maintained as a single‐copy (e.g., IFN‐β), whereas IFN‐HA1 genes frequently underwent multiple‐copy expansion (Figure 4b,c).

All the birds possessed only one type I IFN gene at the HACD4 locus. The sequence identity matrix revealed that this avian IFN‐HA shared significantly greater identity with mammalian IFN‐HA2s than with IFN‐HA1s (*p* < 0.0001; Figure 4d,e). These data suggest that the single‐copy IFN‐HA2 gene represents the ancestral state at HACD4 and that mammalian IFN‐HA1 clusters evolved via lineage‐specific duplications in mammals and reptiles.

### 2.6 Convergent Diversification of Type I IFN Genes at the UBAP2 Locus in Birds

Although chicken IFN‐UBs were initially proposed to be homologs of human IFN‐α/β (Sick et al. 1996), syntenic and phylogenetic analyses revealed that these genes evolved independently from HACD4‐linked type I IFNs (Krause and Pestka 2015; Gan et al. 2018; Chen et al. 2022; Santhakumar, Rubbenstroth, et al. 2017). Unlike the dichotomous IFN‐HA1/HA2 clusters at the HACD4 locus, IFN‐UBs almost formed a single monophyletic group (Figure 2a,b), underscoring the distinct evolutionary history of IFN‐UBs compared with that of IFN‐HAs.

**FIGURE 5:**
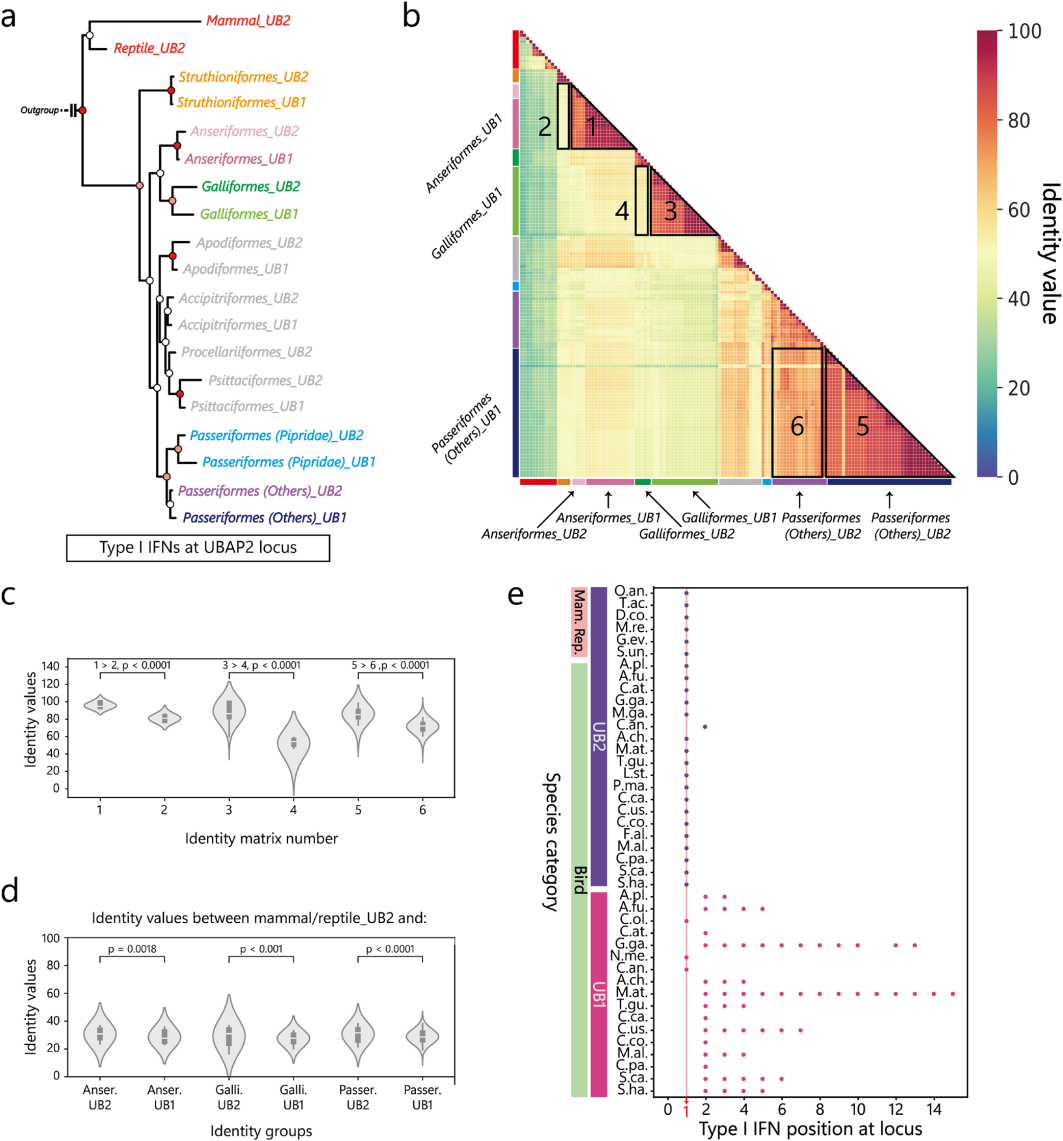
IFN‐UBs diversify into UB1 and UB2. (a) Phylogenetic relationships of all IFN‐UBs. Some IFN taxa are colored gray because of the low‐quality of the genome assembly of the corresponding species. (b) The sequence identity matrix of the phylogenetic tree in (a). The meaning of each color bar corresponds to the taxon of the same color in (a) (i.e., the red bar represents IFN‐UB2 in mammals and reptiles). (c) Violin plots of the identity values in the six regions in (b) and the results of the significance test. (d) Violin plots of identity values between mammalian/reptile IFN‐UB2s and other IFN‐UBs in birds and the results of the significance test. (e) The relative type I IFN position of IFN‐UBs. Each data point represents the position of a type I IFN gene at the UBAP2 locus in the corresponding species.

When phylogenetically analyzed as a separate group, the IFN‐UB genes consistently bifurcated into two distinct subgroups (IFN‐UB1 and IFN‐UB2) within each avian order we examined (Figure 5a). Thus, the type I IFN genes at the UBAP2 locus have been revised to 44 IFN‐UB2 genes and 95 IFN‐UB1 genes (Figures S5, S6). The sequence identity matrix revealed higher identity values among IFN‐UB1s within the same order (regions 1, 3, and 5) compared to those of IFN‐UB1 vs. IFN‐UB2 (regions 2, 4, and 6), and this pattern was consistent across different avian orders (e.g., Anseriformes, Galliformes, and Passeriformes) (*p* < 0.0001; Figure 5b,c). Monotremes and reptiles have a single‐copy IFN‐UB gene, and the sequence identity matrix revealed that this monotreme/reptile IFN‐UB shared significantly greater identity with bird IFN‐UB2s than with IFN‐UB1s (*p* < 0.01; Figure 5d), which proves that they belong to IFN‐UB2 (Figure 5a). Additionally, the IFN‐UB2 genes mostly existed in single‐copy form and were located at the beginning of the IFN‐UB cluster, whereas the IFN‐UB1 genes were present in multiple‐copy form following IFN‐UB2 (Figure 5e).

Although phylogenetically distinct, the IFN‐HA and IFN‐UB followed analogous evolutionary trajectories. Type I IFNs at both loci originated from single‐copy ancestors (IFN‐HA2 at HACD4; IFN‐UB2 at UBAP2) and independently diversified into multicopy subtypes via locus‐specific duplications.

### 2.7 Four‐Cysteine Type I IFNs Are the Ancestor of Amniote Type I IFNs

While Krause et al. proposed IFN‐β as the ancestor of mammalian type I IFNs on the basis of phylogenetic analysis (Krause and Pestka 2015), Secombes and Zou (2017) argued that 4‐cysteine (4C) type I IFNs (e.g., IFN‐κ and IFN‐UB2) represent the ancestral form, given their conservation in fish, amphibians, reptiles, birds, and mammals (Secombes and Zou 2017). However, mammalian IFN‐βs have only 2 conserved cysteines (2C), which is unique among all amniote type I IFNs (Secombes and Zou 2017). The apparent conflict between these competing hypotheses remains unresolved.

IFN‐ν may hold the key to resolving this issue. In this study, we performed a detailed sequence analysis for this understudied type I IFN subtype. Given that IFN‐ν is present only in a subset of species, we have additionally included several species from the order Carnivora in the analysis for this section to provide a sufficient sample of IFN‐ν sequences (Table S7).

Phylogenetic analysis revealed that IFN‐ν formed a distinct monophyletic group within mammals, which is a sister group to the IFN‐β and IFN‐HA2 clades. Additionally, IFN‐ν sequences from the newly added species all clustered within this group (Figure S7a). This provides robust phylogenetic evidence for IFN‐ν as a distinct type I IFN subtype. Furthermore, the sequence identity matrix revealed that sauropsid IFN‐HA2s shared significantly greater identity with these IFN‐νs than with IFN‐βs (*p* < 0.001; Figure S7b,c), which indicates that the divergence between IFN‐ν and IFN‐β was an ancient event that occurred early in mammalian evolution.

**FIGURE 6:**
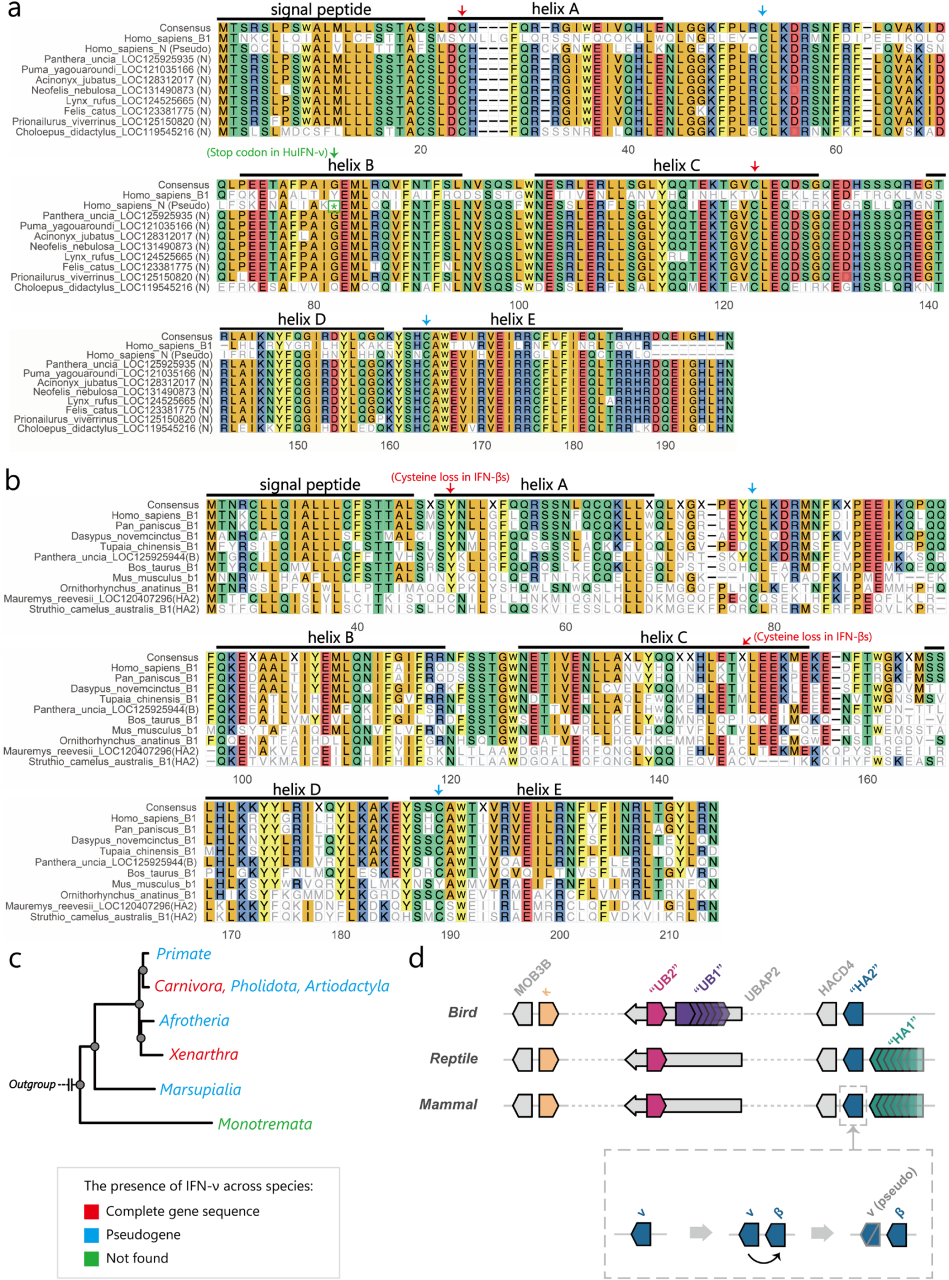
IFN‐ν is the ancestor of mammalian IFN‐HAs (a). The MSA results of IFN‐ν in a representative species, human ( *Homo sapiens* ) IFN‐β, are included to annotate their protein domains. (b) The MSA results of IFN‐β in mammals and IFN‐HA2 in sauropsids. Conserved cysteines in (a) and (b) are indicated by colored arrows, whereas cysteine losses in IFN‐β are indicated by red arrows. (c) The presence of IFNN (encoding IFN‐ν) in mammals. The gene statuses of “complete gene sequence” (colored in red), “pseudogene” (colored in blue), and “not found” (colored in green) are illustrated. (d) A general diagram of the relative patterns of most type I IFN genes in amniotes.

MSA results revealed that all IFN‐ν orthologs (including those of newly added Carnivora species) conservatively retained four‐cysteine (4C) residues, resulting in the formation of two disulfide bonds (Figure 6a). This stands in sharp contrast to IFN‐β orthologs, which exhibited a conserved structure containing only two cysteines (2C), resulting from the specific loss of the pair of cysteines that form the C1–C3 disulfide bond (in fact, the mouse has lost both pairs of cysteines) (Figure 6b).

In contrast to the canonical IFNB‐HACD4 arrangement in most therians, we found a functional IFNN (encoding IFN‐ν) positioned between IFNB and HACD4 in the snow leopard ( *Panthera uncia* ; Figure 4b) and southern two‐toed sloth ( *Choloepus didactylus* ; Figure S5). NCBI database mining revealed complete IFNN in additional Felidae and Phocidae species (Figure 6c), although it is pseudogenized in primates (Krause and Pestka 2005).

On the basis of the results of the above syntenic and phylogenetic analyses, we propose that the 4C form is the ancestor of amniote “intronless” type I IFN at the MOB3B, UBAP2 and HACD4 loci and that IFN‐ν may serve as the ancestor of mammalian IFN‐HAs (Figure 6d).

### 2.8 Interchromosomal Duplication of Type I IFN Genes

**FIGURE 7:**
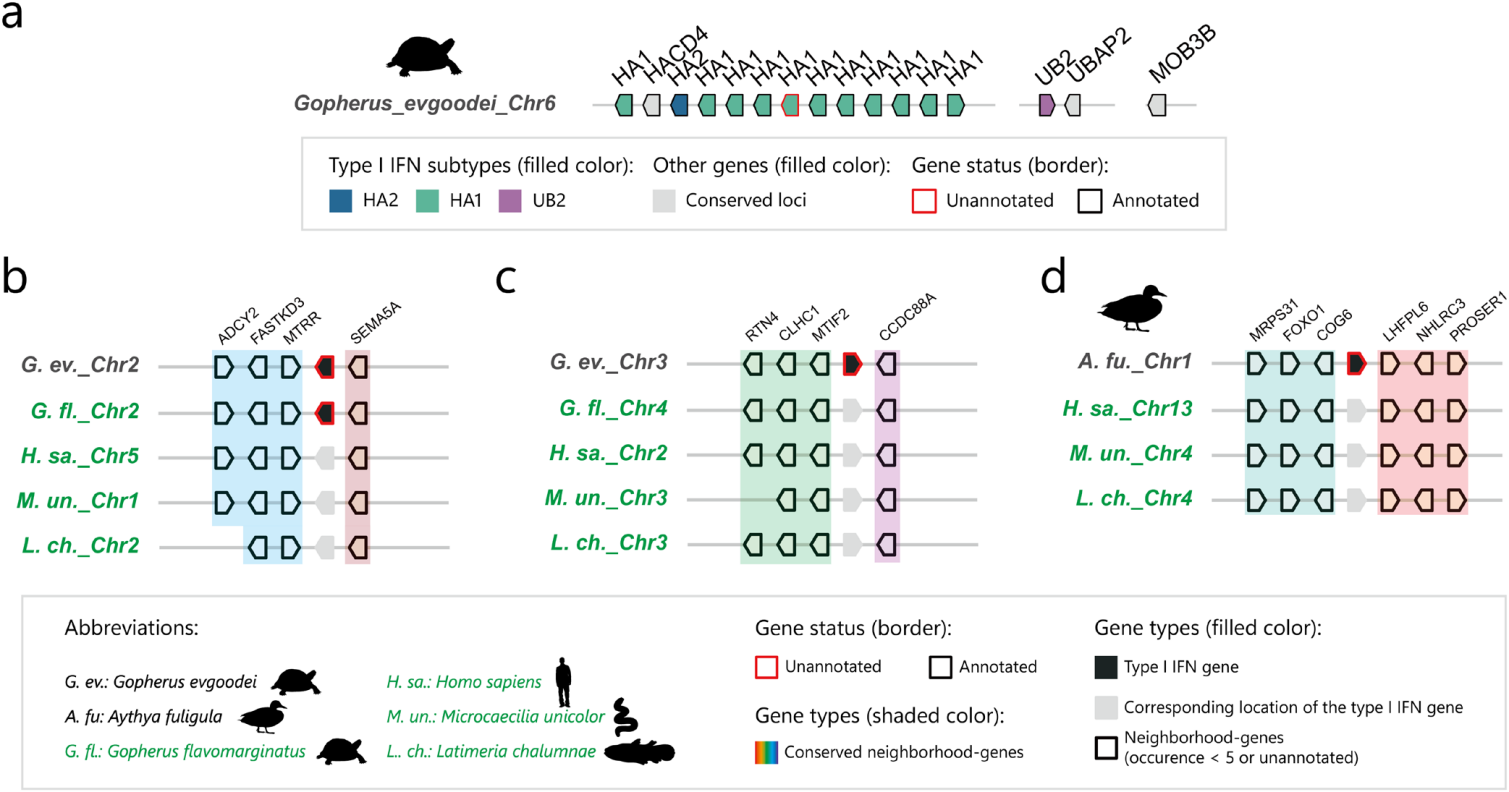
Illustrations of interchromosomal duplication of type I IFN genes in candidate species. (a) Canonical type I IFN distribution on chromosome 6 of Goode's thornscrub tortoise ( *Gopherus evgoodei* ). Gene arrows of type I IFN genes and corresponding conserved loci are differently colored. (b) Interchromosomal gene duplication events on chromosome 2 of Goode's thornscrub tortoise. Type I IFN genes (colored in black) that are not flanked by common neighborhood genes are presented, and conserved neighborhood genes in Bolson tortoise ( *Gopherus flavomarginatus* or *G. fl*.), human ( *Homo sapiens* or *H. sa*.), unstriped caecilian ( *Microcaecilia unicolor* or *M. un*.), and coelacanths ( *Latimeria chalumnae* or *L. ch*.) are marked by different color shadings. (c) Interchromosomal gene duplication events on chromosome 3 of Goode's thornscrub tortoise. (d) Interchromosomal gene duplication events on chromosome 1 of tufted ducks ( *Aythya fuligula* ). Only the first and last type I IFN genes on the locus are presented in (b, c, and d), and the gene orientations are adjusted to align the gene orientations in Goode's thornscrub tortoise in these figures.

Interestingly, we identified 27 type I IFN genes from 7 species located outside the three conserved loci (MOB3B, HACD4, and UBAP2) (Tables S3, S4). Among these genes, 21 type I IFN genes with adjacent genes were syntenic with type I IFN‐flanking genes on human chromosome 9 (Figure S4; Table S3), strongly suggesting that chromosomal rearrangements contributed to their noncanonical genomic distribution. An additional five type I IFN genes were identified in Goode's thornscrub tortoises ( *Gopherus evgoodei* ) and were distributed across chromosomes 1, 2, 3, and 24. In contrast, the canonical type I IFN loci (HACD4, MOB3B, and UBAP2) were located on chromosome 6 in this species (Figure 7a, Table S4). Notably, the tortoise chromosome 2 type I IFN was flanked by MTRR and SEMA5A genes whose orthologs reside on disparate chromosomes in humans (chromosome 5), unstriped caecilian (chromosome 1), and coelacanth (chromosome 2) (Figure 7b). In contrast, the HACD4, MOB3B, and UBAP2 type I IFN loci were located on chromosome 9 (humans), chromosome 2 (caecilians), and chromosome 1 (coelacanths), respectively (Figure 1e). This syntenic inconsistency strongly supports an interchromosomal mechanism for this type I IFN gene duplication. Furthermore, the type I IFN gene on chromosome 2 has also been identified in the Bolson tortoise ( *Gopherus flavomarginatus* ), providing additional support for the above conclusion (Figure 7b). Similarly, orthologs of the MTIF2 and CCDC88A genes flanking the tortoise chromosome 3 type I IFN gene were mapped to human chromosome 2, unstriped caecilian chromosome 3, and coelacanth chromosome 3, which suggests the occurrence of another independent interchromosomal duplication event (Figure 7c). Orthologs of the genes flanking the chromosome 1 type I IFN genes and chromosome 24 type I IFN gene were not located on the same chromosome or even identified in the human, caecilian, or coelacanth genomes, precluding subsequent syntenic analysis (Table S4).

**FIGURE 8:**
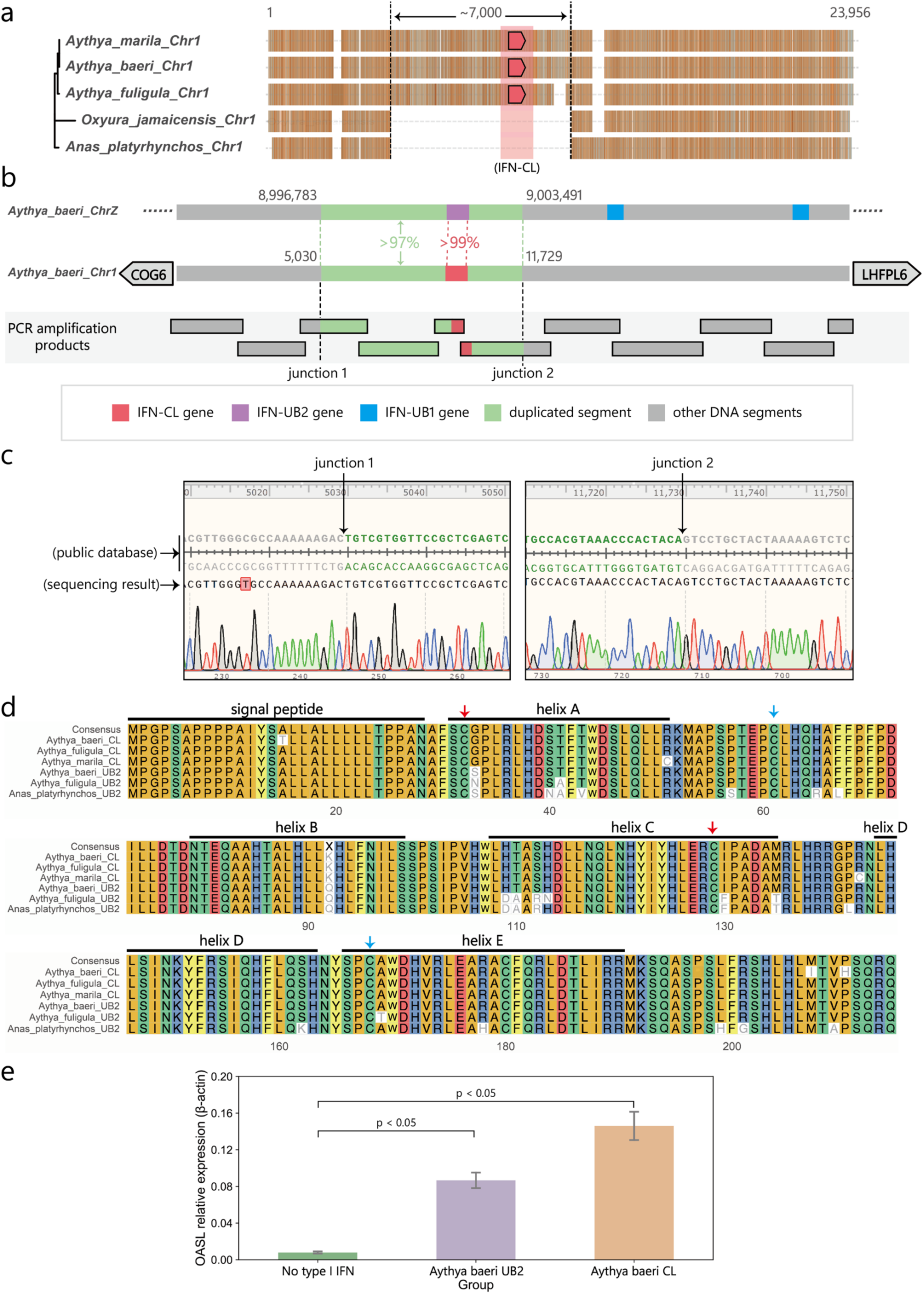
Unique IFN‐CL gene on chromosome 1 of diving ducks is found to be functional. (a) The MSA results of the DNA segment from COG6 to LHFPL6 in diving ducks (*Aythya* spp.), mallards ( *Anas platyrhynchos* ), and ruddy ducks ( *Oxyura jamaicensis* ) in public databases. The numbers above represent the corresponding positions within the segment. (b) The results of PCR amplification and sequencing prove the presence of an IFN‐CL gene in Baer's pochard ( *Aythya baeri* ). Colors are used to denote the following: IFN‐CL gene (red), IFN‐UB2 gene (purple), IFN‐UB1 gene (blue), duplicated segment (green), and other DNA segments (gray). The corresponding PCR fragments are presented below. (c) Both the nucleotide junctions of the inserted segment in chromosome 1 in the public database and the sequencing results. (d) The MSA result of IFN‐CL and IFN‐UB2 in ducks. IFN‐UB2 of *Aythya marila* was not found due to its low‐quality genome assembly. (e) The induction of OASL expression by IFN‐CL and IFN‐UB2 in blood cells from Baer's pochards.

Although avian type I IFN genes were typically restricted to chromosome Z, we identified an exceptional case in tufted ducks ( *Aythya fuligula* ), in which one type I IFN gene (assigned as IFN‐UB2) was localized to chromosome 1 and flanked by COG6 and LHFPL6 (thus referred to as “IFN‐CL,” Figure 7d). Notably, the orthologs of these flanking genes had conserved synteny across distant vertebrates: human chromosome 13, caecilian chromosome 4, and coelacanth chromosome 4 (Figure 7d). Furthermore, type I IFN genes were found at the same locus in three additional diving duck species (*Aythya* spp.) but were absent in both the mallard ( *Anas platyrhynchos* ) and ruddy duck ( *Oxyura jamaicensis* ) (Figure 8a). These findings strongly support that interchromosomal duplication of type I IFN genes occurred on chromosome 1 during the early divergence of diving ducks.

Sequence analysis revealed that an approximately 7 kb DNA segment inserted at this locus had high sequence homology to the region flanking the IFN‐UB2 gene on chromosome Z (99% identity for the type I IFN coding region; 97% identity for the whole segment) (Figure 8b). To confirm this insertion event, we amplified 11 DNA fragments by overlapping PCR using blood samples from Baer's pochards ( *Aythya baeri* ) and sequenced the PCR products. Both insertion boundaries (5′ and 3′ junctions) were unambiguously mapped, which conclusively validated the insertion event (Figure 8c, Figure S8). Consistent with its genomic origin, the IFN‐CL protein sequences were markedly similar to those of diving duck IFN‐UB2s (Figure 8d).

To functionally characterize IFN‐CL, we cloned the coding sequences of Baer's pochard IFN‐CL and IFN‐UB2 into recombinant plasmids and expressed them in HEK293F cells. The supernatants were subsequently used to stimulate peripheral blood mononuclear cells (PBMCs). Both IFN‐CL and IFN‐UB2 significantly upregulated the expression of OASL (Figure 8e), a known interferon‐stimulated gene (ISG) that is specifically induced by type I interferons across multiple species, including ducks. These results demonstrate that IFN‐CL is a functional type I interferon that originated from an interchromosomal duplication event on chromosome 1 of the diving duck.

## 3 Discussion

**FIGURE 9:**
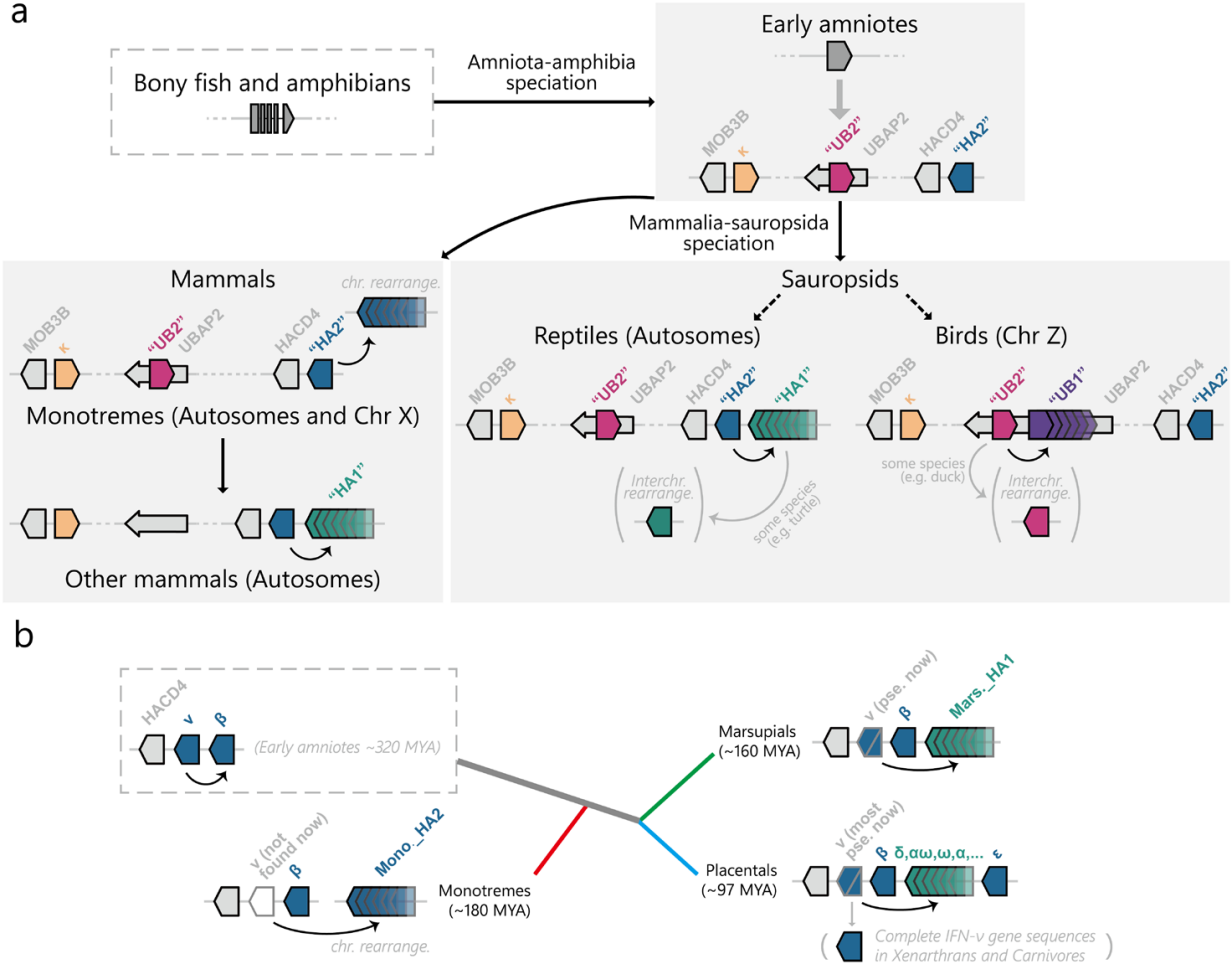
Locus‐specific convergent evolution and interchromosomal rearrangements contribute to the diversification of amniote type I IFNs. (a) Overview of the evolutionary history of amniote type I IFN that illustrates how the single‐copy IFN‐HA2 and IFN‐UB2 genes, located at the ancestral ends of the HACD4 and UBAP2 loci, respectively, independently diversified into multicopy genes. (b) Evolutionary history of mammalian IFN‐HA. The evolutionary pattern of IFN‐ν is clearly demonstrated. Note that the branch lengths and colors in the tree have no evolutionary meaning.

It has been hypothesized that evolutionary retroposition events of intron‐containing type I IFN transcripts gave rise to “intronless” type I IFNs in amniotes during the period when vertebrates adapted their living environment from water to land (Secombes and Zou 2017; Gan et al. 2018; Qi et al. 2010; Chen et al. 2021). To investigate this evolution of amniote type I IFN genes, we developed an analytical pipeline by integrating the GENE‐GRADE algorithm into the IFN‐SCOPE model and iteratively determined the type I IFN conserved loci. The finding that these loci were conserved on the same chromosome across all major amniote lineages leads us to propose that these three type I IFN loci were present in the most recent common ancestor (MRCA) of modern amniotes and that each locus evolved independently (Figure 9). Using synteny‐guided phylogenetic analysis that considers chromosomal localization relative to conserved adjacent genes, position within type I IFN clusters and transcriptional orientation, we revealed the following findings (Figure 9a): (1) Convergent diversification patterns at HACD4 and UBAP2 loci produced single‐copy IFN‐HA2/IFN‐UB2 variants and multicopy IFN‐HA1/IFN‐UB1 expansions; (2) the HACD4‐proximal type I IFN gene (IFN‐HA2 subcluster, specifically IFN‐ν in mammals) represents the ancestral form of this locus; (3) in birds with UBAP2‐locus expansions, the type I IFN gene proximal to the 3′‐end of UBAP2 shows ancestral characteristics; and (4) interchromosomal rearrangement of type I IFN genes was clearly observed in several sauropsid lineages.

Among mammalian type I interferons, IFN‐β has unique characteristics, distinguished by both its induction mechanisms and functional properties (Wittling et al. 2020; Fox et al. 2020; Ng et al. 2016). While phylogenetic analyses initially proposed IFN‐β as the ancestral mammalian type I IFN at the HACD4 locus (Krause and Pestka 2015), our synteny‐guided evolutionary reconstruction identified HACD4‐proximal IFN‐ν, with two evolutionarily conserved disulfide bonds, as the authentic progenitor of mammalian IFN‐HA (Figure 6). The evolutionary pattern of mammals at the HACD4 locus, characterized by pervasive IFN‐ν loss alongside conserved single‐copy IFN‐β, indicates functional overlap between these IFN‐HA2 subtypes (Figure 9b). These findings strongly suggest that precise dosage regulation is critical for their immunological functions. Notably, intact IFNN genes (encoding IFN‐ν) persist in some carnivores (Figure 6b), whereas IFN‐β shows lineage‐specific expansions in ruminants (buffalo, sheep, goats, and deer) (Walker and Roberts 2009; Peters et al. 2020) and selected rodents (Krause and Pestka 2015). These exceptions highlight the need to investigate the mechanisms driving unconventional IFN‐HA2 multiplication in specific lineages.

Our synteny‐guided approach resolves longstanding ambiguity in avian type I IFN evolution: (1) confirming MOB3B‐adjacent genes as genuine IFN‐κ orthologs and (2) establishing the single HACD4‐locus gene as the true avian counterpart to mammalian IFN‐HA2 (IFN‐ν/β/ε). This reclassification (Figures 1, 3 and 4) corrects prior interpretations (Santhakumar, Iqbal, et al. 2017; Gao et al. 2018) and provides a validated evolutionary framework for comparative immunological studies.

Chicken IFN‐UB2 (chIFN‐β) and IFN‐UB1 (chIFN‐α) exhibit distinct biological activities and are considered evolutionary paralogs of their human counterparts (Sick et al. 1996; Schultz et al. 2004). Strikingly, comparative genomic analysis revealed noticeable conservation of cis‐regulatory elements in the IFN‐UB2 promoter across vertebrate lineages, including mammalian IFN‐β and selected type I IFN subtypes, in amphibians and bony fishes (Chen et al. 2021; Sick et al. 1996). This exceptional evolutionary preservation suggests strong selective pressure for maintaining the transcriptional regulation of IFN‐β‐like genes. While the functional dichotomy between human IFN‐β and IFN‐α has been extensively characterized, their distinct biological outcomes are largely attributed to differential receptor binding affinities (Schreiber and Piehler 2015). However, the evolutionary and functional significance of type I IFN diversification in nonmammalian amniotes represents a critical knowledge gap in comparative immunology.

Previous studies have established local gene duplication as a major mechanism of type I IFN diversification in amniotes (Krause and Pestka 2015, 2005). Our work extends this understanding by identifying interchromosomal duplication events in Goode's thornscrub tortoises and tufted ducks (Figure 7). Notably, we discovered an interchromosomal duplication on chromosome 1 during early diving duck radiation that generated IFN‐CL (Figure 8e). Sequence analysis revealed that IFN‐CL represents an interchromosomal duplication of IFN‐UB2, which remains single‐copy in most avian species. This evolutionary innovation in diving ducks holds particular significance given that wild and domestic ducks serve as key reservoirs for pandemic influenza viruses (Webster 2023), and different susceptibilities exist between diving ducks and dabbling ducks (e.g., mallards) (Soda et al. 2023). Thus, how this unconventional duplication of IFN‐CL contributes to susceptibility to influenza or other viral infections should be investigated in the future.

In summary, these findings significantly advance our understanding of amniote type I IFN evolution by demonstrating locus‐specific diversification while providing a framework for predicting type I IFN functions through comparative immunology. Moreover, this work provides an evolutionary blueprint for developing novel type I IFN‐based antiviral therapies.

## 4 Materials and Methods

**FIGURE 10:**
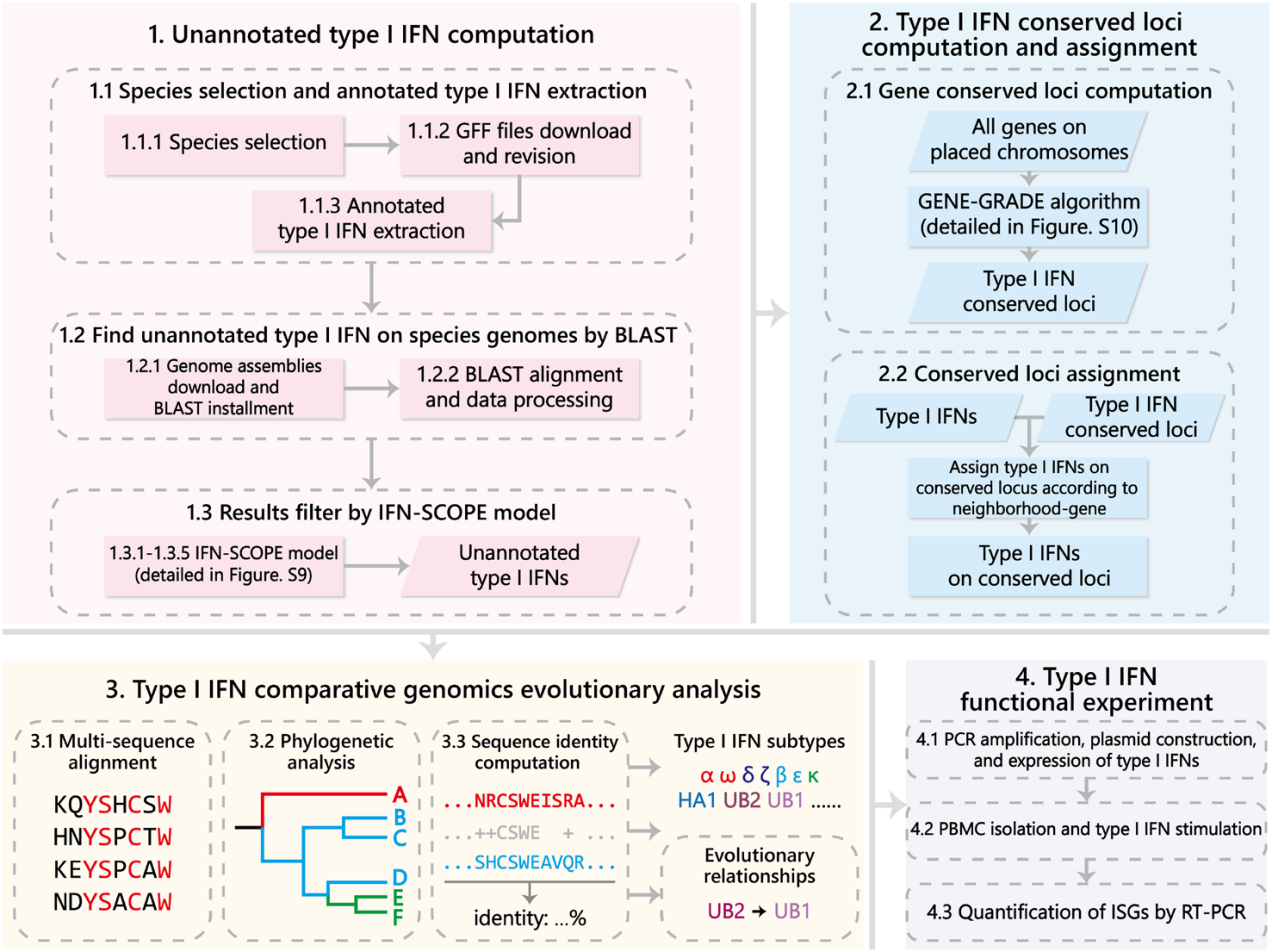
Flowchart of the study. The numerical labels presented to each segment in the figure correspond to the numerical labels in the Materials and Methods section.

The research methods in this paper can be divided into four sections: unannotated type I IFN computation, type I IFN conserved loci computation and assignment, type I IFN comparative genomics evolutionary analysis, and type I IFN functional experiments (Figure 10).

### 4.1 Unannotated Type I IFN Computation

#### 4.1.1 Species Selection and Annotated Type I IFN Extraction

##### 4.1.1.1 Species Selection

We first collected all the orders of amniotes from the NCBI taxonomy database. For those orders for which RefSeq annotation files were available, we selected representative species on the basis of the following principles: species with recently updated RefSeq records, experimental animals, and poultry or livestock. Next, the phylogenetic relationships of our candidate species were presented (Figure S1).

##### 4.1.1.2 Download and Revision of Gff Files

After determining the species of interest for our study, we downloaded all the general feature format (gff) files (Table S2) of these species from the NCBI Assembly database. Afterward, all the gene names in the gff files were comprehensively revised according to their “product,” “description” and “gene abbreviation” terms to reduce the problem of nonstandardization within the database.

##### 4.1.1.3 Annotated Type I IFN Extraction

We filtered and extracted all annotated type I IFN information through the following steps: (1) included genes containing the fields “type I” and “interferon/IFN;” and (2) on the basis of step (1), excluded genes containing the fields “type II,” “type III” or “factor,” “induced/inducible” and “stimulated/stimulator.” Type I IFN “pseudogenes” and “low‐quality proteins” were also excluded from our study (Table S2).

#### 4.1.2 Identification of Unannotated Type I IFN in Species Genomes by BLAST

To identify unannotated type I IFN that was missed by the NCBI Eukaryotic Genome Annotation Pipeline, we used BLAST (Altschul et al. 1990) to find possible protein‐coding regions of type I IFN genes in the genomes of candidate species.

##### 4.1.2.1 Genome Assembly Download and BLAST Installation

We downloaded the genome assemblies of our candidate species from the NCBI Assembly database (Table S2) and installed a Linux version of BLAST+ (v2.13.0).

##### 4.1.2.2 BLAST Alignment and Data Processing

We first constructed a BLAST database using all the genome assemblies (command: *makeblastdb – in genome_assembly_name – dbtype nucl – out database_name*). Afterward, BLAST alignments were carried out by querying all annotated type I IFN against the BLAST databases (command: *tblastn – query IFN_input – db db_name – out alignment_result_name – evalue 1e – 5 – outfmt*). Next, all BLAST alignment result sequences were examined forward and backward (under the open reading frame) along their genomic locations to identify sites that corresponded to the start codon (i.e., ATG) and the stop codon (i.e., TAA, TAG, and TGA), and the corresponding sequences (start codon to stop codon) were saved as the test dataset. They were subsequently entered into the IFN‐SCOPE model to obtain prediction results for unannotated type I IFN.

#### 4.1.3 Filtering of Results by the IFN‐SCOPE Model

##### 4.1.3.1 Data Extraction and Training Dataset Construction

We extracted all the annotated type I IFN (Section 4.1.1.3) and their neighborhood genes from the gff files as the candidate genes for investigation. The IFN‐SCOPE model is detailed in Figure S9. We defined the term “neighborhood genes” as “10” genes (type I IFN genes were excluded) on the same chromosome that were closest to the position of a particular type I IFN gene A ($A_{\text{position}}$), which was previously reported (Williams and Bowles 2004). $A_{\text{position}}$ was determined by Equation (1)

(1) $$A_{\text{position}}=\frac{A_{\text{start}}+A_{\mathrm{end}}}{2}$$

where $A_{\text{start}}$ and $A_{\mathrm{end}}$ denote the start and end sites of gene A on the chromosome, respectively. We subsequently used the amino acid sequences of all type I IFN and their neighborhood genes to construct the training dataset. The amino acid sequences of some type I IFN neighborhood genes could not be successfully accessed or downloaded because of the nonstandardization of the database and were not included in our study.

##### 4.1.3.2 Feature Selection

We performed feature selection (Zhang et al. 2023; Zhang, Dai, et al. 2021; Zhang, Liu, et al. 2021) through the amphiphilic pseudo amino acid composition (APAAC) (Chou 2005) method. A type I IFN amino acid sequence P that is composed of L residues was described by Equation (2)

(2) $$\begin{array}{c} P=R_{1}R_{2}R_{3}R_{4}R_{5}R_{6}\ldots R_{L-1}R_{L} \end{array}$$

where $R_{n}\left(n=1,2,3,\ldots ,L\right)$ denotes the n−th amino acid of P, and $R_{n}=\text{Alanine}\left(A\right),\text{Leucine}\left(L\right),\text{Glycine}\left(G\right)\ldots$. Afterward, we extracted the following 20+2λ‐dimensional feature vector V from P (we adopted λ=30 in this study from a previous report (Chen et al. 2018)), described by Equation (3)

(3) $$\begin{array}{c} V={\left[v_{1},v_{2},\ldots ,v_{20},v_{20+1},\ldots ,v_{20+\lambda },v_{20+\lambda +1},\ldots ,v_{20+2\lambda }\right]}^{T} \end{array}$$

where T is the transpose sign and $v_{u}\left(u=1,2,3,\ldots ,20+2\lambda \right)$ is the u−th feature in V, which was calculated by Equation (4)

(4) $$\begin{array}{c} v_{u}=\left\{\begin{array}{c} \frac{f_{u}}{\sum_{i=1}^{20}f_{i}+w\sum_{j=1}^{2\lambda }\tau_{j}},\;1\leq u\leq 20\\ \frac{w\tau_{u}}{\sum_{i=1}^{20}f_{i}+w\sum_{j=1}^{2\lambda }\tau_{j}},\;20+1\leq u\leq 20+2\lambda \end{array}\right. \end{array}$$

where w is the weight factor (we adopted w=0.05 in this study from a previous report (Chen et al. 2018)), and $f_{i}\left(i=1,2,3,\ldots ,20\right)$ is the normalized occurrence frequency of the i‐th amino acid in P, which reflects the compositional information of the type I IFN amino acid sequence; $\tau_{j}\left(j=1,2,3,\ldots ,2\lambda \right)$ denotes the j−tier hydrophilicity–hydrophobicity factor of P, which reflects the sequence‐related structural information of type I IFN and was calculated by Equation (5).

(5) $$\begin{array}{c} \tau_{j}=\left\{\begin{array}{c} \frac{1}{L-k}\sum_{n=1}^{L-k}{{\text{phobi}}_{n}}^{*}\cdot {{\text{phobi}}_{n+k}}^{*},\;j\;\text{is}\;\mathrm{odd}\\ \frac{1}{L-k}\sum_{n=1}^{L-k}{{\text{phili}}_{n}}^{*}\cdot {{\text{phili}}_{n+k}}^{*},\;j\;\text{is even} \end{array}\right. \end{array}$$

where $k=\left\lceil \frac{j}{2}\right\rceil$ (rounding off of $\frac{j}{2}$), ${{\text{phobi}}_{n}}^{*}$ and ${{\text{phili}}_{n}}^{*}$ denote the normalized hydrophobicity value and normalized hydrophilicity value of the n‐th amino acid $R_{n}$, respectively; these values were obtained from *Z*‐score normalization on the basis of previously reported hydrophobicity and hydrophilicity values (Tanford 1962; Hopp and Wood 1981), which were determined by Equations (6 and 7), respectively. The dot (·) denotes the multiplication sign.

(6) $$\begin{array}{c} {{\text{phobi}}_{n}}^{*}=\frac{{\text{phobi}}_{n}-\mu_{\text{phobi}}}{\sigma_{\text{phobi}}} \end{array}$$

(7) $$\begin{array}{c} {{\text{phili}}_{n}}^{*}=\frac{{\text{phili}}_{n}-\mu_{\text{phili}}}{\sigma_{\text{phili}}} \end{array}$$

where ${\text{phobi}}_{n}$ and ${\text{phili}}_{n}$ denote the hydrophobicity and hydrophilicity values of $R_{n}$, respectively; $\mu_{\text{phobi}}$ and $\mu_{\text{phili}}$ denote the mean values of all ${\text{phobi}}_{n}$ and ${\text{phili}}_{n}$ values, respectively; and $\sigma_{\text{phobi}}$ and $\sigma_{\text{phili}}$ denote the standard deviation of all ${\text{phobi}}_{n}$ and ${\text{phili}}_{n}$ values, respectively.

##### 4.1.3.3 Feature Dimensional Reduction

We performed feature dimension reduction (Gao et al. 2023; Song et al. 2022; Zhang, Zhao, et al. 2021) on the basis of the feature importance of the model (Pedregosa et al. 2011), which aimed to maximize the predictive accuracy of the IFN‐SCOPE model. Specifically, the model was first trained by the initial set of features, and then we obtained the coefficient $\omega_{u}$ for each feature $x_{u}$. Afterward, feature $x_{u}$ was considered unimportant and pruned if the absolute value of its corresponding coefficient $\left|\omega_{u}\right|$ was less than the threshold $\bar{\omega }$ (Equation 8). In this study, we used the SelectFromModel algorithm (Pedregosa et al. 2011) for dimensional reduction.

(8) $$\begin{array}{c} \bar{\omega }=\frac{\sum_{u}\left|\omega_{u}\right|}{u} \end{array}$$

where $\bar{\omega }$ is the average of all $\left|\omega_{u}\right|$, and $u\left(u=1,2,3,\ldots ,80\right)$ is the index of feature $x_{u}$. K‐fold cross‐validation was carried out on the dataset before and after dimensional reduction, and the average $F_{1}$ score (Equation 9) was used to evaluate the performance of feature dimensional reduction.

(9) $$\begin{array}{c} F_{1}-\text{score}=\frac{2\times \text{Precision}\times \text{Recall}}{\text{Precision}+\text{Recall}} \end{array}$$

where Precision and Recall were obtained from Equations (10 and 11), respectively.

(10) $$\begin{array}{c} \text{Precision}=\frac{\mathrm{TP}}{\mathrm{TP}+\mathrm{FP}} \end{array}$$

(11) $$\begin{array}{c} \text{Recall}=\frac{\mathrm{TP}}{\mathrm{TP}+\mathrm{FN}} \end{array}$$

where TP (true positive) is the number of perfectly identified type I IFN, TN (true negative) is the number of perfectly identified type I IFN neighborhood genes, FP (false‐positive) is the number of incorrectly identified type I IFN genes that are actually type I IFN neighborhood genes, and FN (false‐negative) is the number of incorrectly identified type I IFN neighborhood genes that are actually type I IFN genes.

##### 4.1.3.4 Model Construction and Training

We used logistic regression (Wu et al. 2020; Lei et al. 2020; Zhang et al. 2019; Zhang et al. 2017) as the classifier of our model (Figure S8). By adopting L2 regularization, logistic regression reduced the complexity of the coefficients and thus prevented the model from overfitting. Specifically, we used feature vectors as the input, which were the output of the feature dimensional reduction procedure, and used the sample labels (is/not type I IFN) as the output. Our logistic regression was optimized by a stochastic average gradient descent algorithm.

##### 4.1.3.5 Model Evaluation and Prediction

We used the $F_{1}$ score (Equation 9), Accuracy (Equation 12) and area under ROCcurve (Equation 13) to evaluate the performance of the IFN‐SCOPE model.

(12) $$\begin{array}{c} \text{Accuracy}=\frac{\mathrm{TN}+\mathrm{TP}}{\mathrm{TN}+\mathrm{FN}+\mathrm{TP}+\mathrm{FP}} \end{array}$$

(13) $$\begin{array}{c} \mathrm{ROC}\;\text{curve}=\left\{\begin{array}{c} x:\mathrm{FPR}=\frac{\mathrm{FP}}{\mathrm{FP}+\mathrm{TN}}\\ y:\mathrm{TPR}=\frac{\mathrm{TP}}{\mathrm{TP}+\mathrm{FN}} \end{array}\right. \end{array}$$

In Equation (13), FPR and TPR denote the false‐positive rate and true positive rate, respectively, of the model under different thresholds. The well‐trained IFN‐SCOPE model was subsequently used to predict the label for each sample in the test dataset (Section 4.1.3.1). Samples with positive predictions were saved as unannotated type I IFN after double‐filtering and reviewed by annotated type I IFN and were finally designated “*species_chromosome_position*” (such as *Anas_platyrhynchos_NC_051804.1_POS71719872*) (Table S2).

### 4.2 Type I IFN Conserved Loci Computation and Assignment

#### 4.2.1 Computation of Conserved Gene Loci

The degree centrality algorithm (Ghasemi et al. 2014; You et al. 2022) indicates that the node with the maximum degree (highest number of direct connections) is the most important one. In this study, we developed the GENE‐GRADE algorithm to look for the conserved loci within type I IFN evolution of all genes for our candidate species, which consisted of two parts: knowledge graph construction and conserved loci computation. We describe the GENE‐GRADE algorithm in Figure S10.

##### 4.2.1.1 Knowledge Graph Construction

We only consider genes that are in the revised GFF files and placed on the chromosome of our candidate species during knowledge graph (Wang et al. 2023; Ma et al. 2022; Zhang, Zhang, et al. 2021) construction (Section 4.1.1). Then genes are added into knowledge graph G as individual nodes $N_{\text{gene}}$, and merged IFN‐I nodes which are adjacent to the gene position (Section 4.1.1.2) in G. Next, we build up the gene chains by merging IFN‐I nodes $N_{\mathrm{IFN}}$ and IFN‐I neighborhood genes $N_{\mathrm{nb}\_\text{gene}}$ according to their gene position. Finally, we remove all nodes that are not placed on gene chains.

##### 4.2.1.2 Conserved Loci Computation

We merged all $N_{\mathrm{nb}\_\text{gene}}$ that had the same name for all the gene chains in G. Afterward, the degree centrality algorithm was used to look for the node with the maximum degree. If the degree of $N_{\max\_\text{degree}}$ was > 15, the gene of $N_{\max\_\text{degree}}$ was output and saved as a conserved locus, after which we removed all the gene chains that included $N_{\max\_\text{degree}}$ in G; otherwise, we concluded that the gene of $N_{\max\_\text{degree}}$ existed only in some of our candidate species, which contradicted the meaning of the term “conserved,” and the GENE‐GRADE algorithm ended.

#### 4.2.2 Conserved Loci Assignment

After the conserved loci of type I IFN evolution were determined by the GENE‐GRADE algorithm, we assigned type I IFN genes to their corresponding conserved loci according to the definition of neighborhood genes (Section 4.1.3.1). If one type I IFN gene could not be assigned to any conserved locus, we assigned it to the “exception” category.

The visualization of all conserved loci, gene orientations, numbers, and neighborhood genes of type I IFN genes was implemented by the ggplot2 and gggenes packages in R, as well as seaborn in Python. Specifically, we adopted the following principles to present our results. First, if the nomenclature of a type I IFN in the public database was consistent with our analysis results, the name was displayed above its gene arrow; otherwise, the name displayed inside the parentheses represented the nomenclature from our results, whereas the name displayed outside the parentheses represented the nomenclature in the public database (Figures S5, S6). Second, the relative positions of gene arrows at the same conserved locus were determined on the basis of the position (Section 4.1.3.1) of type I IFN genes.

### 4.3 Type I IFN Comparative Genomics Evolutionary Analysis

#### 4.3.1 Multisequence Alignment

We carried out multisequence alignment (MSA) in this study after inputting the .fasta files of interest into MAFFT (v7.520) (command line: *mafft – – auto – – reorder – – leavegappyregion input_filename > output_filename*). The visualization of the results was implemented by the ggplot2 and ggmsa packages in R. In sequence identity matrices, species categories that corresponded to the taxon in the phylogenetic tree were colored. The ends of the MSA result were trimmed for a clearer presentation.

#### 4.3.2 Phylogenetic Analysis

Phylogenetic trees of type I IFN genes were constructed by neighbor‐joining algorithm through the MSA (Section 4.3.1) results of interest by MEGA, as indicated by previous studies in the field (Krause and Pestka 2015; Chen et al. 2021, 2022; Xu et al. 2013). All the trees of the type I IFN genes were tested by bootstrapping (1500 trials), with the results indicated by the color of the clade nodes (red for bootstrap value>90, pink for 90>bootstrap value>70, and white for bootstrap value<70). We adopted interleukin‐10 (IL‐10) proteins from candidate species as outgroups of type I IFN trees in this study. Phylogenetic trees of species were constructed through TimeTree (Kumar et al. 2022).

The visualization of all phylogenetic results was implemented by the ggplot2, ggtree, and ggstar packages in R. Specifically, we adopted the following principles for assigning or giving nomenclature to unnamed type I IFN in trees. First, for previously reported type I IFN from placentals, we designated all type I IFNs within the same monophyletic group on the basis of the previous name of their subtype (such as α, ω, or β); for previously unreported type I IFN that was present mainly in stem mammals, birds, and reptiles, we proposed new nomenclatures (such as HA2, HA1, UB2, and UB1) on the basis of evidence from synteny, sequence homology, and copy number. Low‐quality type I IFN nodes that were not grouped with any other lineages were pruned for clarity (Table S5).

#### 4.3.3 Sequence Identity Computation

We determined the identity values between all the amino acid sequences of type I IFNs through ClustalO. All the amino acid sequences were first input (command line: *– – infile input_filename – – threads 8 – – MAC‐RAM 8000 – – verbose – – full – – outfmt clustal – – resno – – outfile output_filename – – output – order tree – order – – seqtype protein*) and then converted to the .csv format to assess the identity values between the amino acid sequences. The visualization of the identity matrix results was implemented using Seaborn in Python.

### 4.4 Type I IFN Functional Experiments

#### 4.4.1 PCR Amplification, Plasmid Construction, and Expression of Type I IFNs

We extracted DNA from the anticoagulated blood of Baer's pochards ( *Aythya baeri* ) with a TIANamp Genomic DNA Kit (DP304; Tiangen). Next, we designed primers (Table S6) to carry out PCR amplification and sequencing analysis for the Baer's pochard IFN‐CL gene and its flanking sequences, which included a 22,432 bp DNA segment from COG6 to LHFPL6. The gene fragments of IFN‐CL and IFN‐UB2 were subsequently cloned and inserted into a PTT3 vector with a C‐terminal His tag. All the plasmids were transfected into HEK293F cells, which were subsequently cultured in a constant temperature shaker with 8% CO_2_ at 37°C. After 96 h of culture, the supernatant was collected by centrifugation for 10 min and used for quantitative Western blot analysis.

#### 4.4.2 PBMC Isolation and Type I IFN Stimulation

PBMCs from Baer's pochards were prepared from anticoagulated blood with a duck peripheral blood lymphocyte isolation kit (LTS1090D; TBD Sciences). The cells were maintained in RPMI 1640 medium (Thermo Scientific) supplemented with 10% FBS at 37°C with 5% CO_2_. Next, PBMCs were stimulated with IFN‐CL and IFN‐UB2 for 6 h.

#### 4.4.3 Quantification of ISGs by RT‐PCR

The stimulated cells were collected and washed in ice‐cold PBS and then pelleted and lysed with 0.5 mL of TRIzol (15596026CN; Invitrogen). Total RNA from cells was extracted, treated with RQ1 RNase‐Free DNase (M6101; Promega), and reverse transcribed in 25 μL reactions using M‐MLV Reverse Transcriptase (M1701; Promega) according to the manufacturer's instructions. To quantify the transcript levels, 20 μL reactions were set up, which contained 1 μL of reverse transcription product, 2× PowerUp SYBR Green Master Mix (A25742; Applied Biosystems), and 250 nM forward and reverse primers (Table S6) of glyceraldehyde 3‐phosphate dehydrogenase (GAPDH) or 2′‐5′‐oligoadenylate synthetase like (OASL). The following cycling conditions were used for the QuantStudio Q6 (Applied Biosystems) system: 10 min at 95°C, followed by 40 cycles of 15 s at 95°C and 1 min at 60°C.

## Funding

This work was supported by National Science and Technology Major Project, 2021YFF1201200, 2024ZD0532900. National Natural Science Foundation of China, 62372316. SichuanScience and Technology Program key project 2024YFHZ0091, 2025YFHZ0066.

## Conflicts of Interest

The authors declare no conflicts of interest.

## Supporting information


**Figure S1:** Overview of the phylogenetic relationships of all candidate species in this study.
**Figure S2:** The distribution of all type I IFN genes at conserved loci or “exception” category in mammals.
**Figure S3:** The distribution of all type I IFN genes at conserved loci or “exception” category in birds and reptiles.
**Figure S4:** The presence of type I IFN neighborhood genes that on conserved loci or “exception” category of our candidate species.
**Figure S5:** Proposed novel nomenclature of type I IFN in mammals of our candidate species.
**Figure S6:** Proposed novel nomenclature of type I IFN in birds and reptiles of our candidate species.
**Figure S7:** The primitive origin of single‐copy IFN‐β, IFN‐ν, and IFN‐HA2 gene at the HACD4 locus.
**Figure S8:** The comparisons and identities of DNA sequence in public database and by sequencing.
**Figure S9:** The flowchart of the IFN‐SCOPE model.
**Figure S10:** The flowchart of GENE‐GRADE algorithm.
**Table S1:** Feature dimension, F1‐score, accuracy, and area under ROC curve (AUC) before and after feature dimensional reduction.
**Table S2:** Data sources and computation results in this paper.
**Table S3:** Type I IFN gene with neighborhood genes that are located on the chromosome 9 of human.
**Table S4:** Type I IFN gene with neighborhood genes that are NOT located on the chromosome 9 of human.
**Table S5:** Amniote type I IFNs that cannot be accurately assigned into specific subtypes.
**Table S6:** PCR primers used in this paper.
**Table S7:** Type I IFNs from the following animals were additionally introduced in the analysis of IFN‐ν.

## Data Availability

We develop a Web server to collect the data used in this study. Annotated IFN‐I are accessible at https://interferon.netlify.app/queryInterferon, unannotated IFN‐I are accessible at https://interferon.netlify.app/download, and the genome versions of the species used for BLAST analysis are available at https://interferon.netlify.app/dataVersion. Code Availability: Related codes of data downloading, preprocessing, BLAST, IFN‐SCOPE model, and GENE‐GRADE algorithm are available at Github (https://github.com/FuboMa/DoctorProjects/tree/master/Project%20IFN‐I%20Evolve).
