# Supplementary material for: Locus‐Specific Convergent Evolution and Interchromosomal Rearrangements Contribute to the Diversification of Amniote Type I Interferons
Source: Evol Appl. 2026 May 14;19(5):e70258. doi: 10.1111/eva.70258 (PMC13176653; doi:10.1111/eva.70258)
Supplement: Supplementary file 1 — Figure S1: Overview of the phylogenetic relationships of all candidate species in this study. Figure S2: The distribution of all type I IFN genes at conserved loci or “exception” category in mammals. Figure S3: The distribution of all type I IFN genes at conserved loci or “exception” category in birds and reptiles. Figure S4: The presence of type I IFN neighborhood genes that on conserved loci or “exception” category of our candidate species. Figure S5: Proposed novel nomenclature of type I IFN in mammals of our candidate species. Figure S6: Proposed novel nomenclature of type I IFN in birds and reptiles of our candidate species. Figure S7: The primitive origin of single‐copy IFN‐β, IFN‐ν, and IFN‐HA2 gene at the HACD4 locus. Figure S8: The comparisons and identities of DNA sequence in public database and by sequencing. Figure S9: The flowchart of the IFN‐SCOPE model. Figure S10: The flowchart of GENE‐GRADE algorithm. Table S1: Feature dimension, F1‐score, accuracy, and area under ROC curve (AUC) before and after feature dimensional reduction. Table S2: Data sources and computation results in this paper. Table S3: Type I IFN gene with neighborhood genes that are located on the chromosome 9 of human. Table S4: Type I IFN gene with neighborhood genes that are NOT located on the chromosome 9 of human. Table S5: Amniote type I IFNs that cannot be accurately assigned into specific subtypes. Table S6: PCR primers used in this paper. Table S7: Type I IFNs from the following animals were additionally introduced in the analysis of IFN‐ν. [file EVA-19-e70258-s001.docx]

**Locus-specific Convergent Evolution and Interchromosomal Rearrangements Contribute to the Diversification of Amniote Type I Interferons**

# Supplementary Information

**Supplementary Tables**

**Supplementary Table 1.** Feature dimension, F_1_-score, accuracy, and area under ROC curve (AUC) before and after feature dimensional reduction.

**Supplementary Table 2.** Data sources and computation results in this paper.

**Supplementary Table 3.** Type I IFN gene with neighborhood genes that are located on the chromosome 9 of human.

**Supplementary Table 4.** Type I IFN gene with neighborhood genes that are NOT located on the chromosome 9 of human.

**Supplementary Table 5.** Amniote type I IFNs that cannot be accurately assigned into specific subtypes.

**Supplementary Table 6.** PCR primers used in this paper.

**Supplementary Table 7.** Type I IFNs from the following animals were additionally introduced in the analysis of IFN-ν.

**Supplementary Figures**

Supplementary Figure 1. Overview of the phylogenetic relationships of all candidate species in this study.

**Supplementary Figure 2.** The distribution of all type I IFN genes at conserved loci or “exception” category in mammals.

**Supplementary Figure 3.** The distribution of all type I IFN genes at conserved loci or “exception” category in birds and reptiles.

**Supplementary Figure 4.** The presence of type I IFN neighborhood-genes that on conserved loci or “exception” category of our candidate species.

**Supplementary Figure 5.** Proposed novel nomenclature of type I IFN in mammals of our candidate species.

**Supplementary Figure 6.** Proposed novel nomenclature of type I IFN in birds and reptiles of our candidate species.

**Supplementary Figure 7.** The primitive origin of single-copy IFN-β, IFN-ν, and IFN-HA2 gene at the HACD4 locus.

**Supplementary Figure 8.** The comparisons and identities of DNA sequence in public database and by sequencing.

Supplementary Figure 9. The flowchart of the IFN-SCOPE model.

**Supplementary Figure 10.** The flowchart of GENE-GRADE algorithm.

### Supplementary Table 1. Feature dimension, F_1_-score, accuracy, and area under ROC curve (AUC) before and after feature dimensional reduction.

| **Metrics** | **Before dimensional reduction** | **After dimensional reduction** |
| --- | --- | --- |
| Feature numbers | 80 | 36 |
| F_1_-score | 0.9816 | 0.9830 |
| Accuracy | 0.9901 | 0.9908 |
| AUC | 0.9934 | 0.9945 |

### Supplementary Table 2. Data sources and computation results in this paper.

Data sources include whole genome assemblies, gff files, type I IFN gene and protein sequence data (annotated type I IFNs), and data version for candidate species; computation results include details of unannotated type I IFNs.

| **Data type** | **Data name** | **Download/Analysis link** |
| --- | --- | --- |
| Data sources | Whole genome assemblies | https://www.ncbi.nlm.nih.gov/assembly/ |
|  | gff files | https://www.ncbi.nlm.nih.gov/assembly/ |
|  | Annotated type I IFNs | https://interferon.netlify.app/download |
|  | Data version | https://interferon.netlify.app/dataVersion |
| Computation results | Unannotated type I IFNs | https://interferon.netlify.app/download |

### Supplementary Table 3. Type I IFN gene with neighborhood genes that are located on the chromosome 9 of human.

*H.sa.: Homo sapiens; M.un.: Microcaecilia unicolor; L.ch.: Latimeria chalumnae*.

| **Species** | **Gene name** | **Chromosome** | **Gene category** | **Neighborhood-gene** | | **Neigh. gene on *H.sa.* chromosome** | | **Neigh. gene on *M.un.* chromosome** | | **Neigh. gene on *L.ch.* chromosome** | |
| --- | --- | --- | --- | --- | --- | --- | --- | --- | --- | --- | --- |
|  |  |  |  | **L** | **R** | **L** | **R** | **L** | **R** | **L** | **R** |
| *Trichosurus vulpecula* | LOC118831614 | Chr9 | HA1 | MTAP | SYK | Chr9 | Chr9 | Chr2 | Chr2 | Chr1 | Chr1 |
| *Trichosurus vulpecula* | LOC118831668 | Chr9 | HA1 | MTAP | SYK | Chr9 | Chr9 | Chr2 | Chr2 | Chr1 | Chr1 |
| *Trichosurus vulpecula* | LOC118831665 | Chr9 | HA1 | MTAP | SYK | Chr9 | Chr9 | Chr2 | Chr2 | Chr1 | Chr1 |
| *Trichosurus vulpecula* | LOC118831684 | Chr9 | HA1 | MTAP | SYK | Chr9 | Chr9 | Chr2 | Chr2 | Chr1 | Chr1 |
| *Ornithorhynchus anatinus* | IFN4 | ChrX5 | HA2 | TBC1D2 | TRIM41 | Chr9 | Chr5 | Chr2 | No gene | Chr1 | No gene |
| *Ornithorhynchus anatinus* | IFN3 | ChrX5 | HA2 | TBC1D2 | TRIM41 | Chr9 | Chr5 | Chr2 | No gene | Chr1 | No gene |
| *Ornithorhynchus anatinus* | IFNK | ChrX5 | HA2 | TBC1D2 | TRIM41 | Chr9 | Chr5 | Chr2 | No gene | Chr1 | No gene |
| *Ornithorhynchus anatinus* | LOC114807941 | ChrX5 | HA2 | TBC1D2 | TRIM41 | Chr9 | Chr5 | Chr2 | No gene | Chr1 | No gene |
| *Ornithorhynchus anatinus* | IFN1 | ChrX5 | HA2 | TBC1D2 | TRIM41 | Chr9 | Chr5 | Chr2 | No gene | Chr1 | No gene |
| *Ornithorhynchus anatinus* | LOC114807967 | ChrX5 | HA2 | TBC1D2 | TRIM41 | Chr9 | Chr5 | Chr2 | No gene | Chr1 | No gene |
| *Tachyglossus aculeatus* | LOC119948206 | ChrX4 | HA2 | TRIM41 | TBC1D2 | Chr5 | Chr9 | No gene | Chr2 | No gene | Chr1 |
| *Tachyglossus aculeatus* | LOC119948164 | ChrX4 | HA2 | TRIM41 | TBC1D2 | Chr5 | Chr9 | No gene | Chr2 | No gene | Chr1 |
| *Tachyglossus aculeatus* | LOC119947822 | ChrX4 | HA2 | TRIM41 | TBC1D2 | Chr5 | Chr9 | No gene | Chr2 | No gene | Chr1 |
| *Tachyglossus aculeatus* | LOC119947800 | ChrX4 | HA2 | TRIM41 | TBC1D2 | Chr5 | Chr9 | No gene | Chr2 | No gene | Chr1 |
| *Mauremys reevesii* | LOC120407278 | Chr6 | HA1 | SYK | MTAP | Chr9 | Chr9 | Chr2 | Chr2 | Chr1 | Chr1 |
| *Mauremys reevesii* | LOC120407297 | Chr6 | HA1 | SYK | MTAP | Chr9 | Chr9 | Chr2 | Chr2 | Chr1 | Chr1 |
| *Mauremys reevesii* | LOC120407300 | Chr6 | HA1 | SYK | MTAP | Chr9 | Chr9 | Chr2 | Chr2 | Chr1 | Chr1 |
| *Mauremys reevesii* | LOC120407541 | Chr6 | HA1 | SYK | MTAP | Chr9 | Chr9 | Chr2 | Chr2 | Chr1 | Chr1 |
| *Mauremys reevesii* | LOC120407302 | Chr6 | HA1 | SYK | MTAP | Chr9 | Chr9 | Chr2 | Chr2 | Chr1 | Chr1 |
| *Gopherus evgoodei* | LOC115653518 | Chr6 | HA1 | MTAP | KIAA2026 | Chr9 | Chr9 | Chr2 | Chr2 | Chr1 | Chr1 |
| *Corvus moneduloides* | NC_045511.1_POS26566131 | ChrZ | κ | CAST | C9ORF72 | Chr5 | Chr9 | Chr2 | Chr2 | Chr1 | Chr1 |

### Supplementary Table 4. Type I IFN gene with neighborhood genes that are NOT located on the chromosome 9 of human.

*H.sa.: Homo sapiens; M.un.: Microcaecilia unicolor; L.ch.: Latimeria chalumnae*.

| **Species** | **Gene name** | **Chromosome** | **Gene category** | **Neighborhood gene** | | **Neigh. gene on *H.sa.* chromosome** | | **Neigh. gene on *M.un.* chromosome** | | **Neigh. gene on *L.ch.* chromosome** | |
| --- | --- | --- | --- | --- | --- | --- | --- | --- | --- | --- | --- |
|  |  |  |  | **L** | **R** | **L** | **R** | **L** | **R** | **L** | **R** |
| *Gopherus evgoodei* | NC_044345.1_POS12357003 | Chr24 | HA1 | CADM3 | No gene | Chr1 | No gene | Chr14 | No gene | Chr21 | No gene |
| *Gopherus evgoodei* | NC_044322.1_POS221191147 | Chr1 | HA1 | EPHA1 | YBX3 | Chr7 | Chr12 | Chr14 | Chr14 | No gene | No gene |
| *Gopherus evgoodei* | NC_044322.1_POS221199478 | Chr1 | HA1 | EPHA1 | YBX3 | Chr7 | Chr12 | Chr14 | Chr14 | No gene | No gene |
| *Gopherus evgoodei* | NC_044323.1_POS129589677 | Chr2 | HA1 | MTRR | SEMA5A | Chr5 | Chr5 | Chr1 | Chr1 | Chr2 | Chr2 |
| *Gopherus evgoodei* | NC_044324.1_POS198858452 | Chr3 | HA1 | MTIF2 | CCDC88A | Chr2 | Chr2 | Chr3 | Chr3 | Chr3 | Chr3 |
| *Aythya fuligula* | LOC116501035 | Chr1 | UB2 | COG6 | LHFPL6 | Chr13 | Chr13 | Chr4 | Chr4 | Chr4 | Chr4 |

### Supplementary Table 5. Amniote type I IFNs that cannot be accurately assigned into specific subtypes.

Due to the limitations of the quality of genomic data, three amniote type I IFN genes cannot be accurately assigned since they do not group with any other type I IFN.

| **Species** | **Conserved loci** | **Gene name** |
| --- | --- | --- |
| *Oxyura jamaicensis* | “exception” category | NW_023312189.1_POS988 |
| *Oxyura jamaicensis* | “exception” category | NW_023312179.1_POS7562 |
| *Rhinolophus ferrumequinum* | HACD4 | NC_046295.1_POS18678887 |

### Supplementary Table 6. PCR primers used in this paper.

Because the genome of baer's pochard has not been annotated during experimentation and manuscript preparation, the primers of corresponding genes are designed individually based on BLAST results.

| **Gene name** | **Forward (5’ to 3’)** | **Reverse (5’ to 3’)** |
| --- | --- | --- |
| IFN-CL | CCCCACCTTGTCGTTGTCCAGC | CCCAGCCAATCTATCTATCCAG |
|  | ATCTTCAAACGCTTGGAGTTACAT | GCTTTATACCTGAGCACAGAGCC |
|  | TTCCCAGCAAAGCCAATAACAT | GCAGTAGGAGAACAACGAGCAGT |
|  | ATGACCAACCTTCTGGCGATGC | CCTCTGGATTCCCTTGTATGTG |
|  | ACTCCCTTAGGTTCCATTGAAGA | CGAGGTGGTAGATGTAGTGGTTGA |
|  | CCTCCTCAAACACCTCTTCAACATC | GTCCAGCAACCTGAACTGTAAACAC |
|  | TTTCTGCCATGTTTCCTGTGAA | AGCATCAGCATTTGTTGCCTTC |
|  | GCTTCTTTCTACTTCTGTCCCATAC | TCATAACTACCCTATTCCAGTCCTAA |
|  | GCCAGGGAAGGCAGCCTCACAT | CACCTCCTCAGAGCAGAGCAATAACAAA |
|  | GGCGTTTGATCCAGTCTGATTT | GATTTCCTCCTCCTGCTCCTTC |
|  | CATGTTCCCGGACTGTAAAAGA | CAAAGCGACTGTCCCCTTGGTC |
| GAPDH | CGTGTGGTGGACTTGATGGT | GAGGAGTGGGGGAGACAGAA |
| OASL | AGAATCGGCTCCAAGAGTGC | ATCGTAGGTGGGCAGGATGT |

**Supplementary Table 7.** **Type I IFNs from the following animals are additionally introduced in the analysis of IFN-ν.**

| **Species (scientific name)** | **Species (common name)** |
| --- | --- |
| *Felis catus* | Cat |
| *Panthera leo* | Lion |
| *Puma yagouaroundi* | Jaguarundi |
| *Puma concolor* | Cougar or mountain lion |
| *Panthera pardus* | Leopard |
| *Neofelis nebulosa* | Clouded leopard |
| *Acinonyx jubatus* | Cheetah |
| *Lynx rufus* | Bobcat |
| *Prionailurus viverrinus* | Fishing cat |
| *Panthera onca* | Jaguar |
| *Canis lupus familiaris* | Dog |
| *Ailuropoda melanoleuca* | Giant panda |
| *Mustela putorius furo* | Ferret |


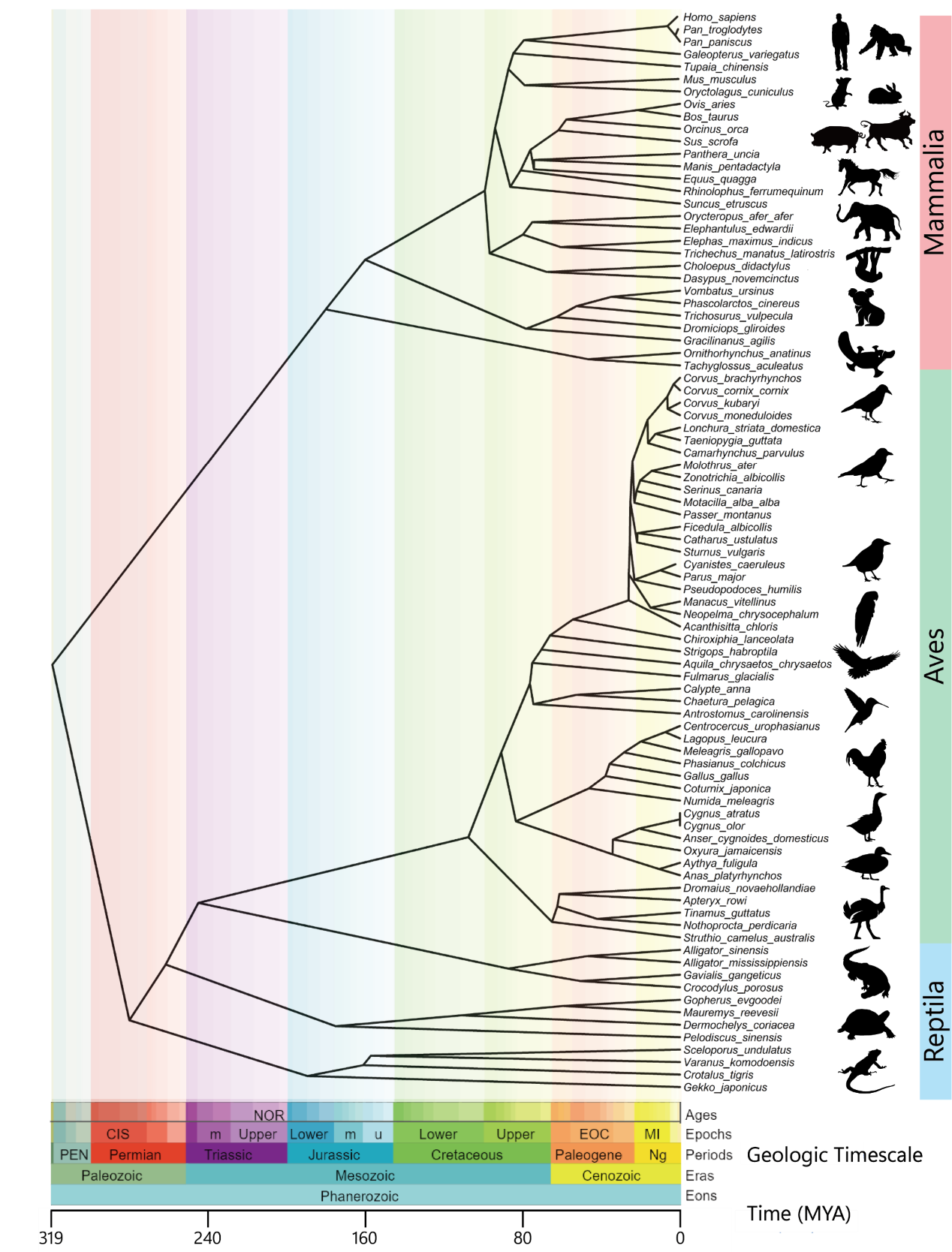


### **Supplementary Figure 1. Overview of the phylogenetic relationships of all candidate species in this study.**

According to the taxonomy of amniotes, candidate mammals (Mammalia, above), birds (Aves, middle) and reptiles (Reptilia, below) are presented in the Figure. The geological timescales and estimated divergence times are provided at the bottom of the Figure. The Figure is curated from TimeTree (Kumar, et al., 2022) and silhouette icons are obtained from Flaticon.


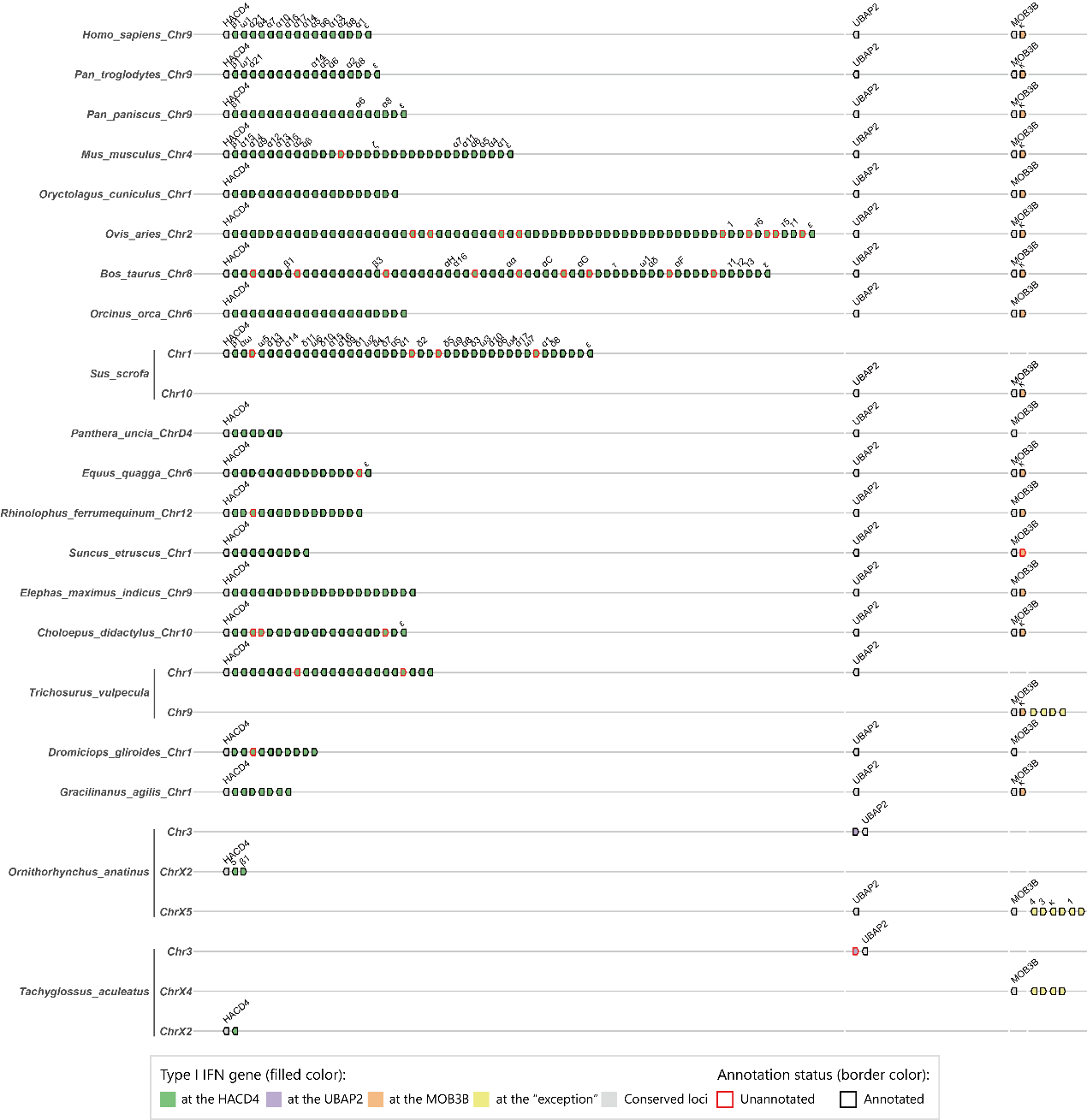


### Supplementary Figure 2. The distribution of all type I IFN genes at conserved loci or “exception” category in mammals.

Type I IFN genes on the chromosomes (letter or number after the scientific name) of mammals are displayed according to their corresponding conserved loci.

**
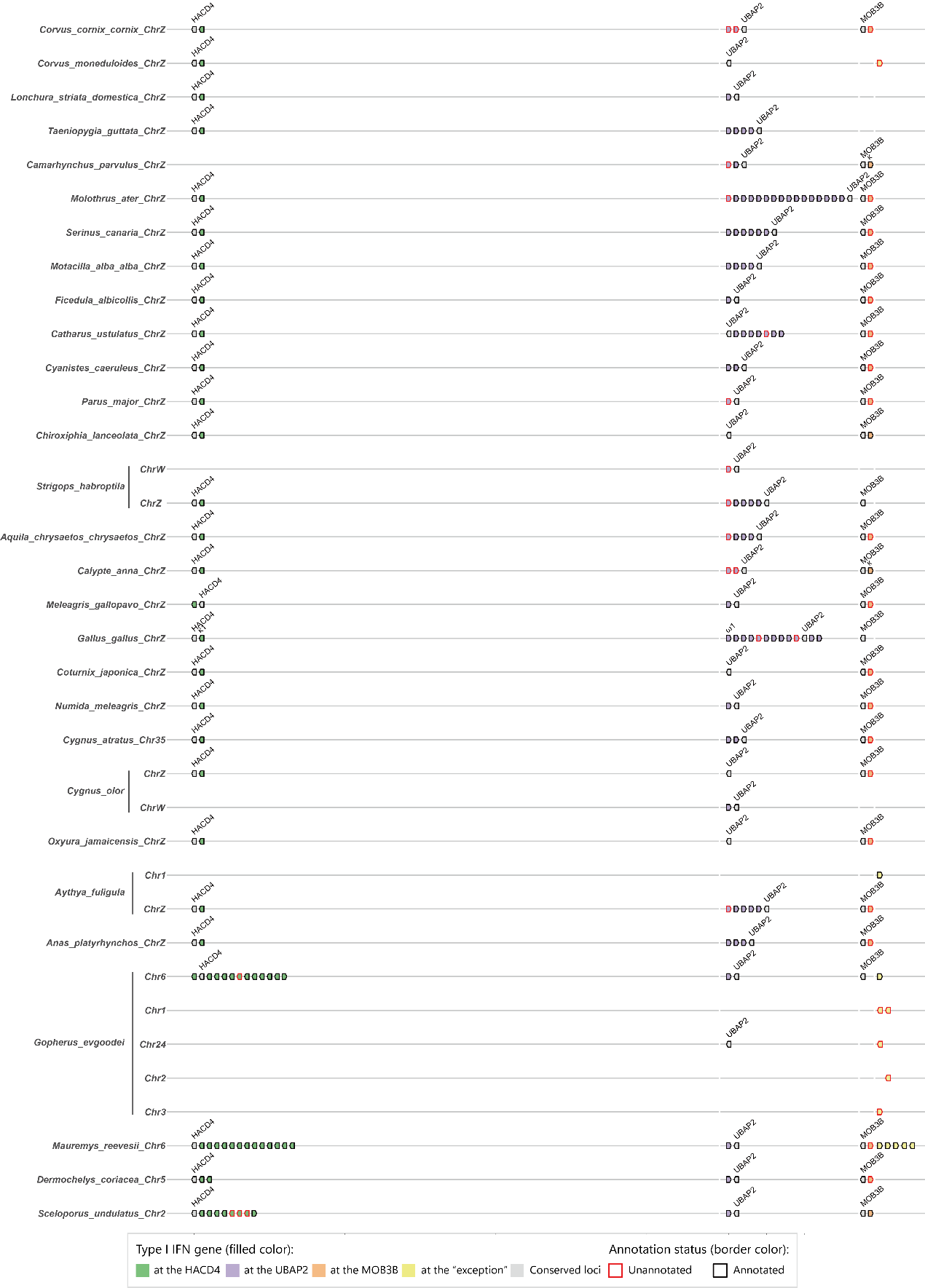
**

### Supplementary Figure 3. The distribution of all type I IFN genes at conserved loci or “exception” category in birds and reptiles.

Type I IFN genes on the chromosomes (letter or number after the scientific name) of birds and reptiles are displayed according to their corresponding conserved loci.


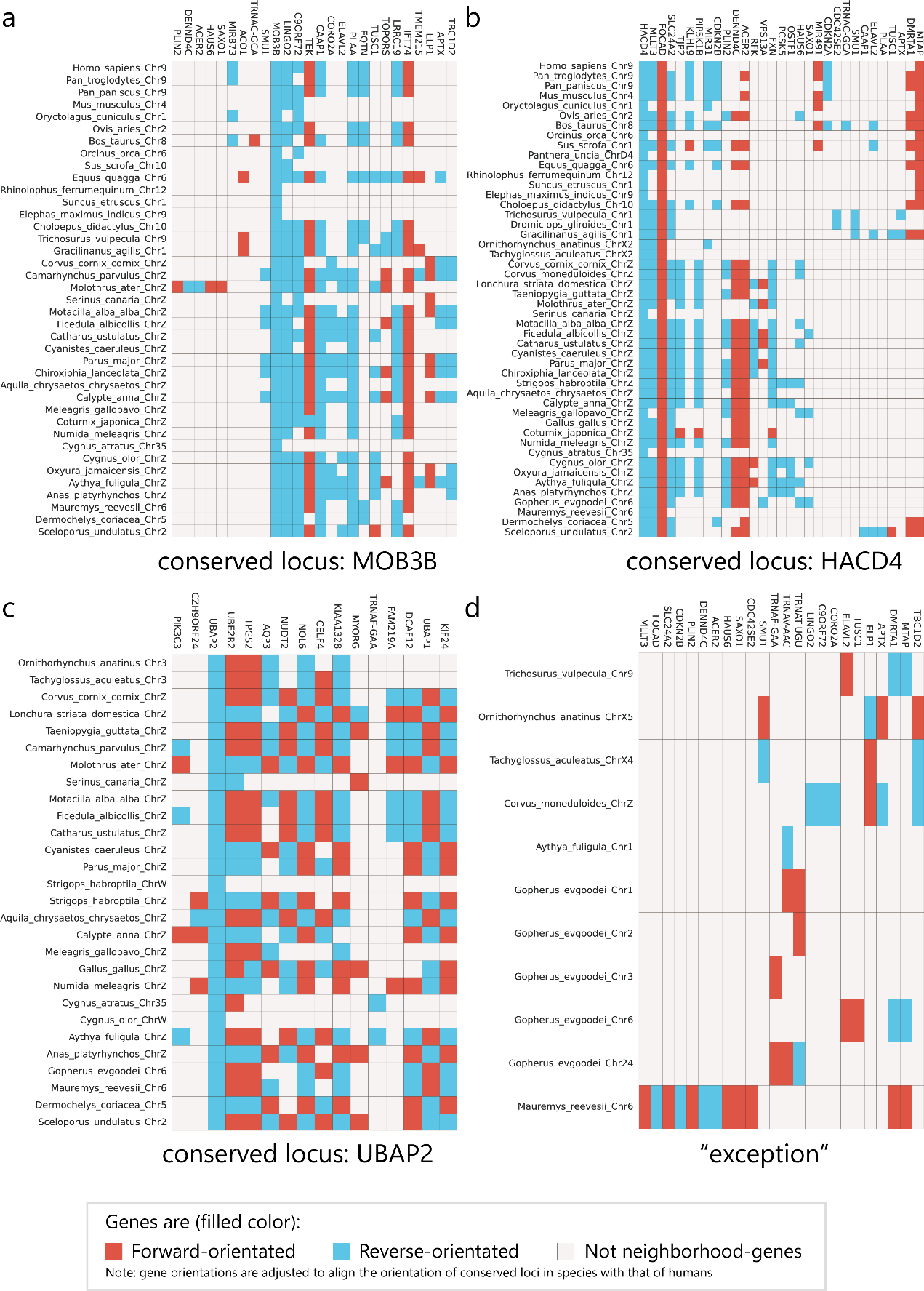


### Supplementary Figure 4. The presence of type I IFN neighborhood-genes that on conserved loci or “exception” category in our candidate species.

In this Figure, neighborhood-gene of type I IFNs (the X-axis) at the MOB3B locus **(a)**, HACD4 locus **(b)**, UBAP2 locus **(c)**, and “exception” category **(d)** of chromosomes (the Y-axis) are accordingly displayed and colored by their gene orientation in each subplot. Only neighborhood-genes occurring more than five times are presented in the figure.


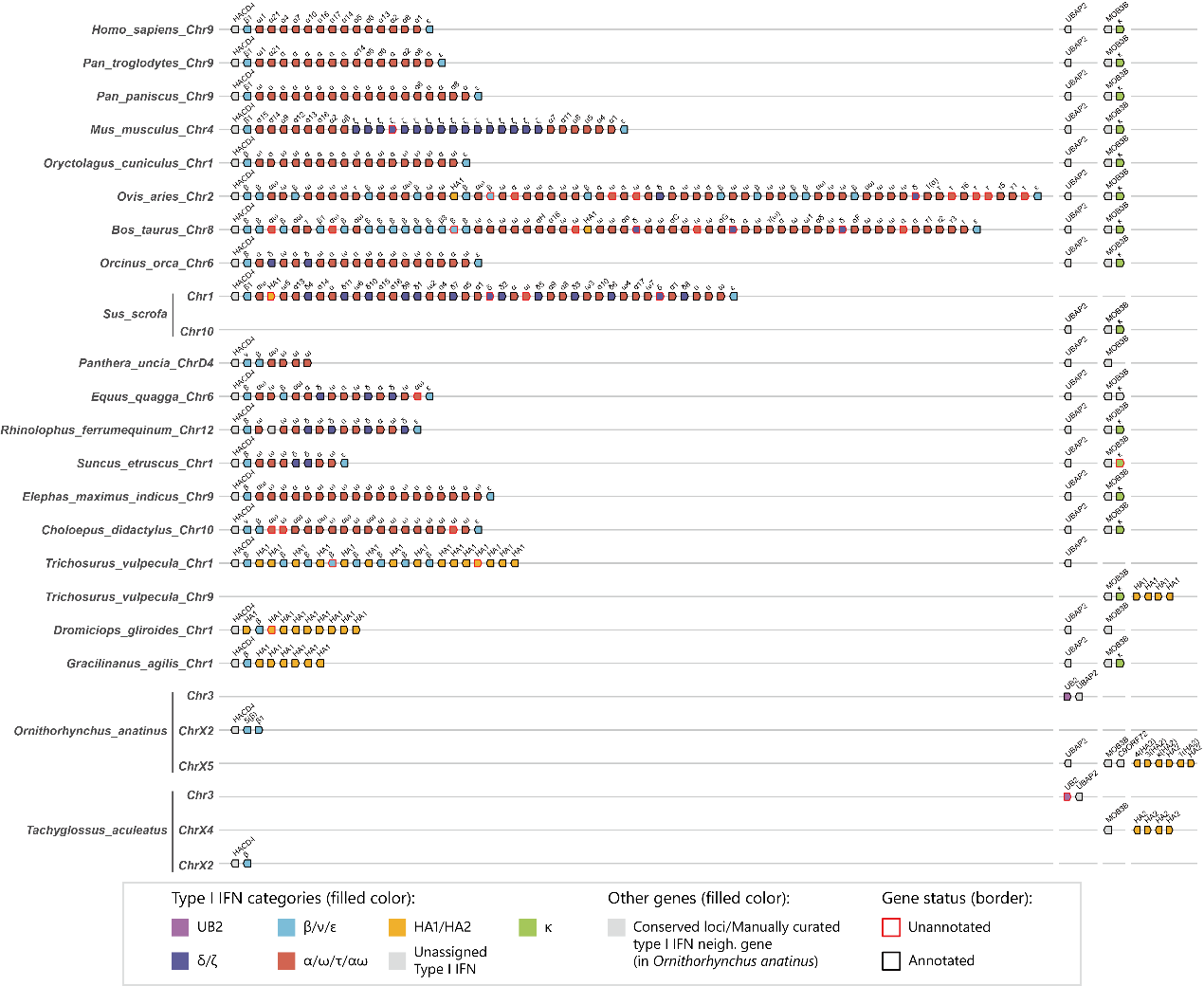


### Supplementary Figure 5. Proposed novel nomenclature of type I IFN in mammals of our candidate species.

The color of each type I IFN gene indicates the proposed novel nomenclature in this study. We have highlighted C9ORF72 in platypus separately for illustrative purpose in the main text.


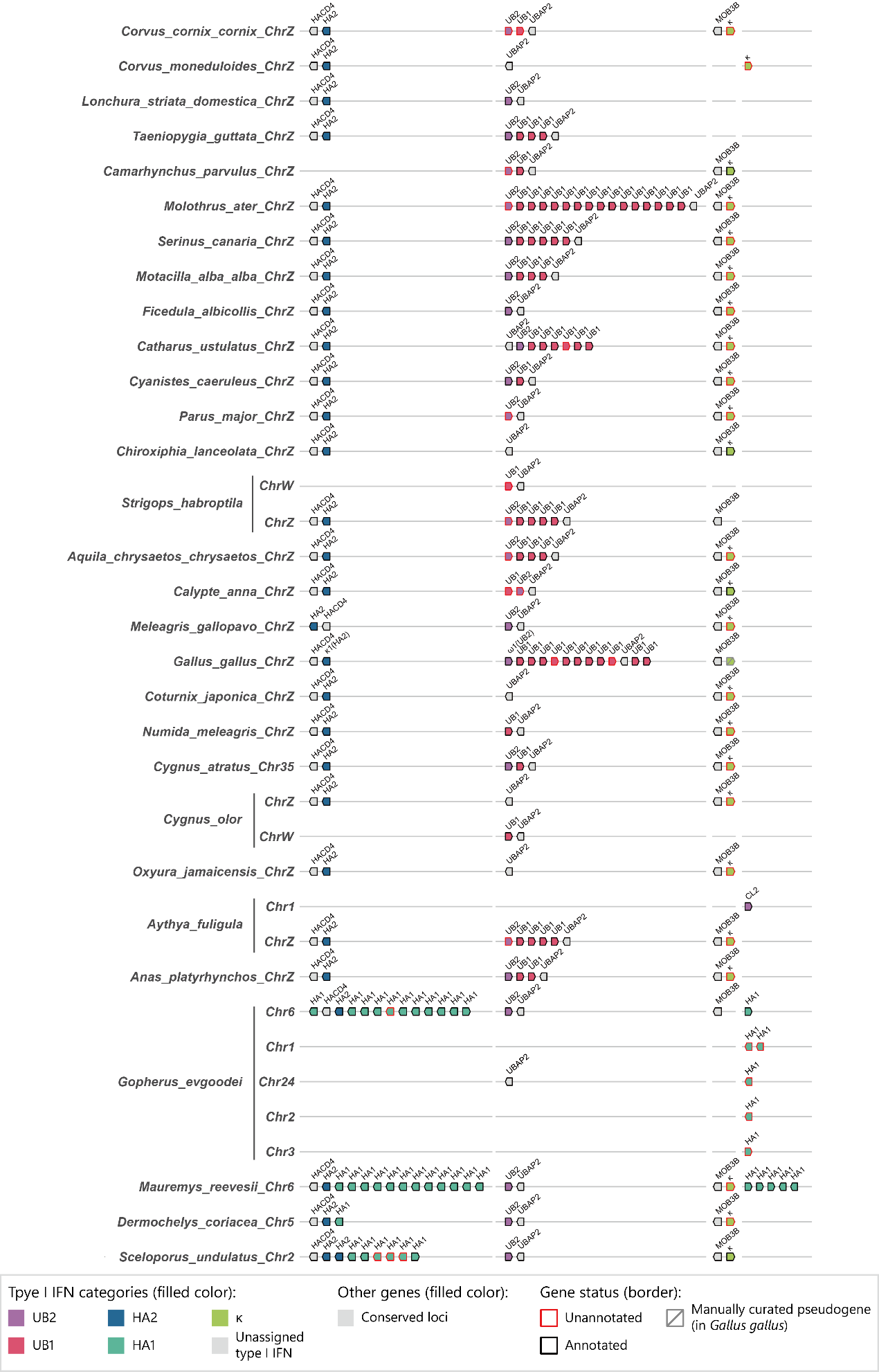


### Supplementary Figure 6. Proposed novel nomenclature of type I IFN in birds and reptiles of our candidate species.

The color of each type I IFN gene indicates the proposed novel nomenclature in this study. We have highlighted the pseudo gene of IFN-κ in chicken (*Gallus gallus*) separately for illustrative purpose in the main text.


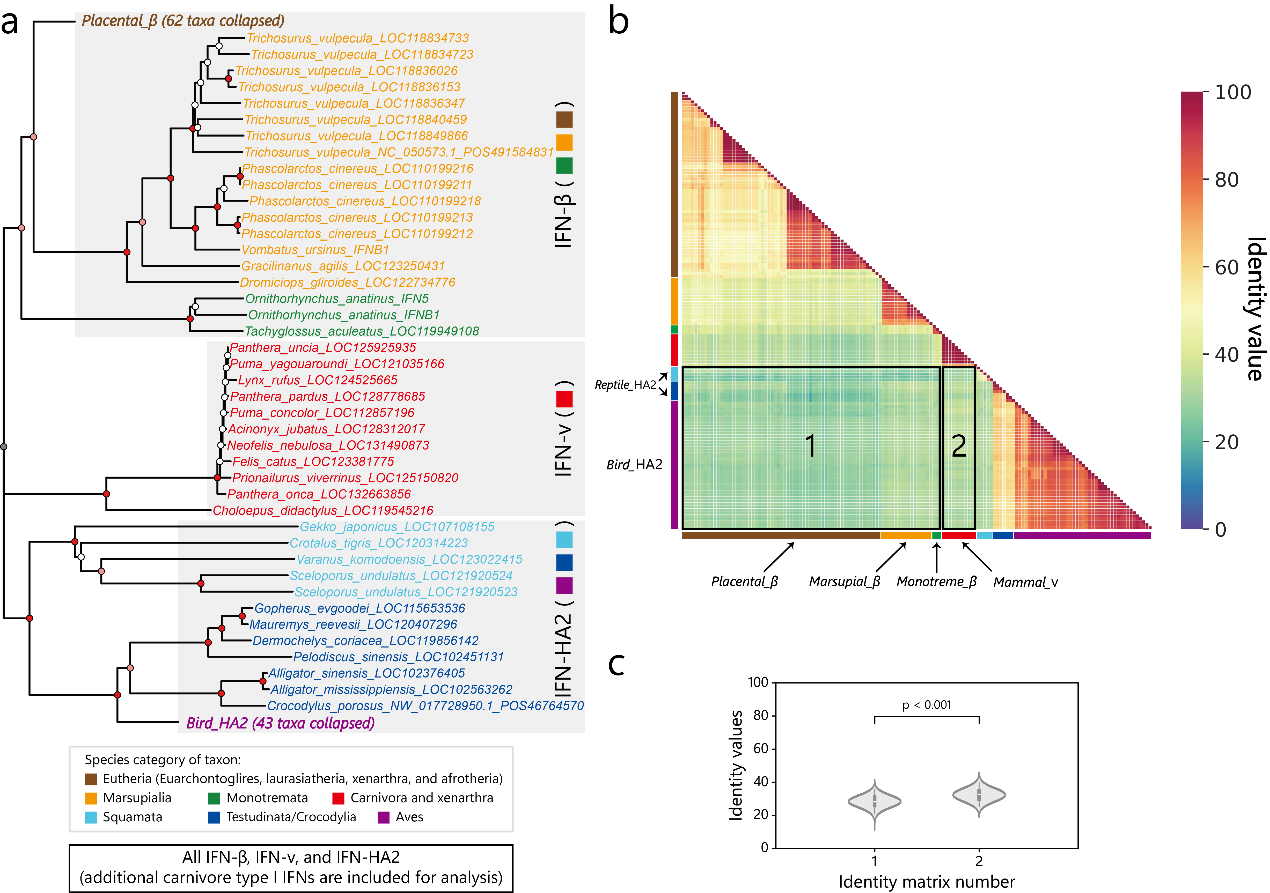


**Supplementary Figure 7. The primitive origin of single-copy IFN-ν at the HACD4 locus.**

**(a)** The primitive origin of single-copy IFN-ν gene at the HACD4 locus. **(b)** The sequence identity matrix of IFN-β, IFN-ν and IFN-HA2 gene in amniotes. The grouping of taxa is indicated by color bars. **(c)** Violin plots of identity values in the two regions in **(b)** and the significance test results.

**
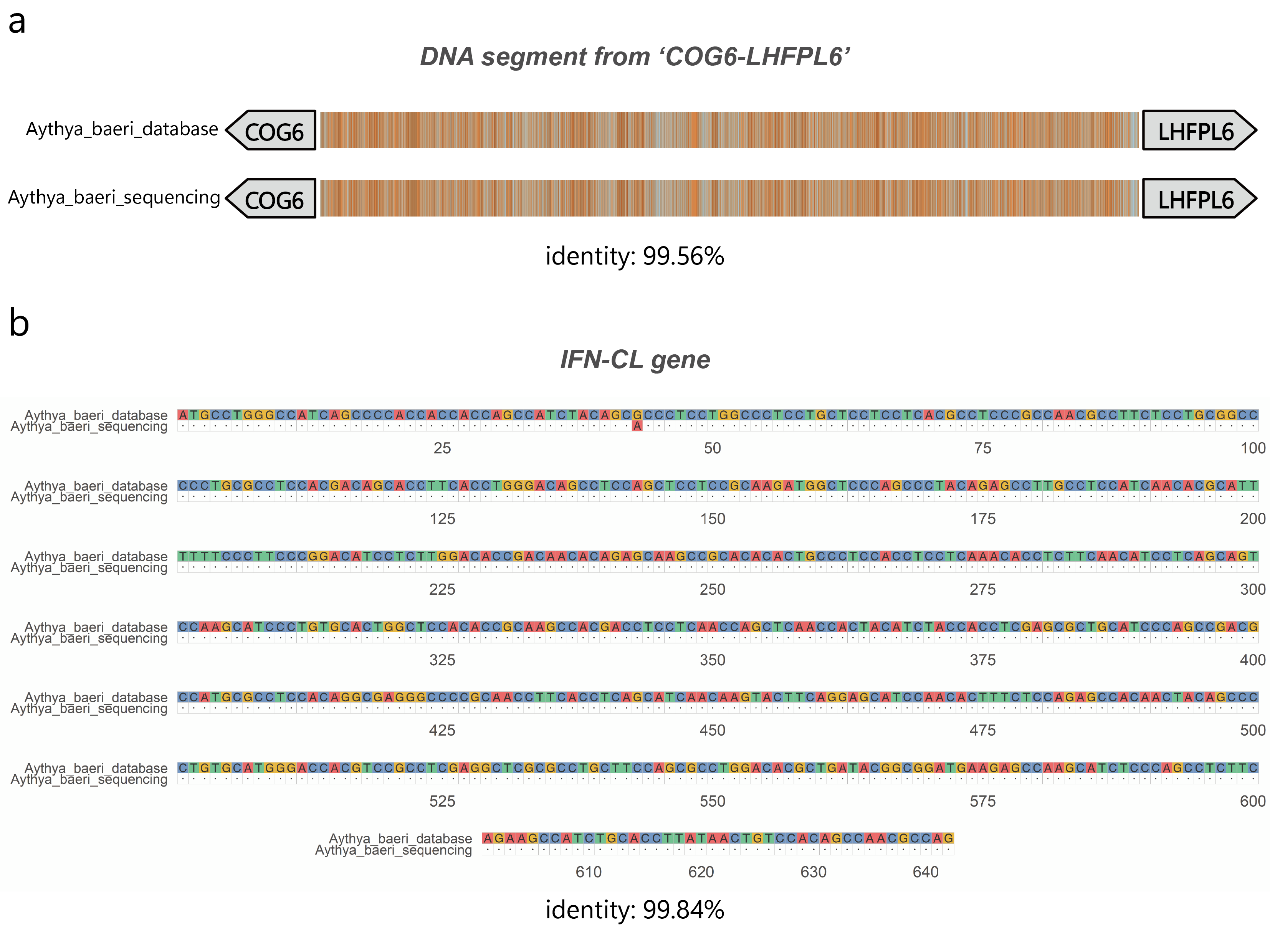
**

### Supplementary Figure 8. The comparisons and identities of DNA sequence in public database and by sequencing.

**(a)** The DNA segment that from COG6 to LHFPL6. **(b)** The IFN-CL gene.

**
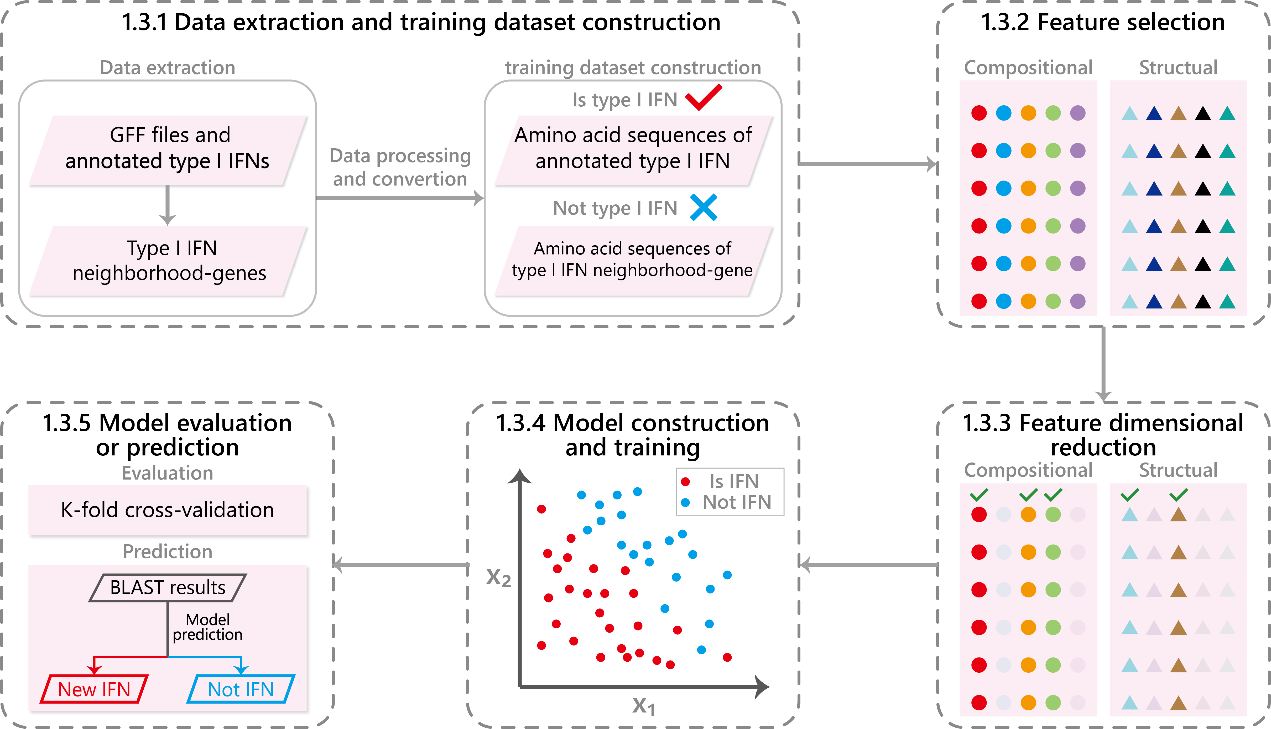
**

### **Supplementary Figure 9. The flowchart of the IFN-SCOPE model.**


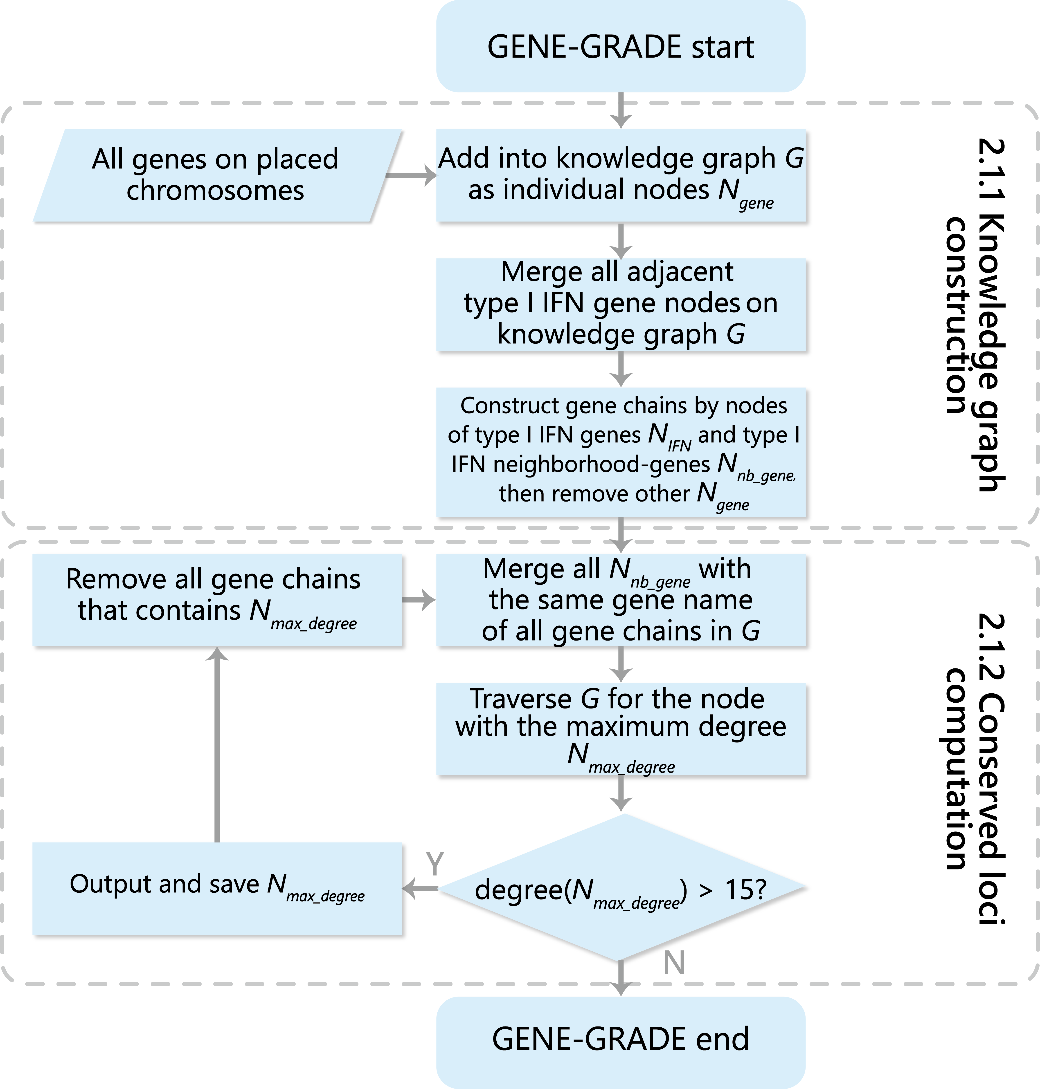


### Supplementary Figure 10. The flowchart of GENE-GRADE algorithm.
